# Supplementary material for: Evidence on COVID-19 Mortality and Disparities Using a Novel Measure, COVID excess mortality percentage: Evidence from Indiana, Wisconsin, and Illinois
Source: PLoS One. 2024 Jan 31;19(1):e0295936. doi: 10.1371/journal.pone.0295936 (PMC10829977; doi:10.1371/journal.pone.0295936)
Supplement: S1 File — (DOCX) [file pone.0295936.s001.docx]

**Appendix for**

**Evidence on COVID-19 Mortality and Disparities Using a Novel Measure,**

**COVID Excess Mortality Percentage: Evidence from Indiana, Wisconsin, and Illinois**

Vladimir Atanasov

William & Mary, Mason School of Business

Natalia Barreto

University of Illinois, Champaign-Urbana

Lorenzo Franchi

Northwestern University

Jeffrey Whittle

Medical College of Wisconsin

John Meurer

Medical College of Wisconsin

Benjamin W. Weston, MD, MPH

Medical College of Wisconsin

Qian (Eric) Luo

George Washington University

Andy Ye Yuan*

Northwestern University, Pritzker School of Law

Ruohao Zhang

Pennsylvania State University

Bernard Black

Northwestern University, Pritzker School of Law and Kellogg School of Management

Draft November 2023

Northwestern University, Pritzker School of Law

Law and Economics Research Paper No. 22-16

Northwestern University, Institute for Policy Research

Working Paper 22-45

*This Appendix can be downloaded without charge from SSRN from:*

<http://ssrn.com/abstract=4244039>

*The paper can be downloaded without charge from SSRN at:*

<http://ssrn.com/abstract=4244054>

* Corresponding author. [andyyuan@law.northwestern.edu](mailto:andyyuan@law.northwestern.edu). Author order follows medical journal conventions, with principal credit to first, second, and last author.

**Appendix for**

**Evidence on COVID-19 Mortality and Disparities Using a Novel Measure,**

**COVID Excess Mortality Percentage: Evidence from Indiana, Wisconsin, and Illinois**

Vladimir Atanasov^b^, Natalia Barreto^e^, Jeff Whittle^c^, John Meurer^c^, Benjamin W. Weston, MD, MPH^c^, Qian (Eric) Luo^d^, Andy Ye Yuan, JD, PhD^a^, Ruohao Zhang^f^, and Bernard Black^a,1^

^a^Northwestern University; ^b^William & Mary; ^c^Medical College of Wisconsin; ^d^George Washington University; ^e^University of Illinois, Urbana-Champaign; ^f^ Pennsylvania Sate University

**Abstract:** This Appendix provides additional data source and methods details and additional results for Atanasov et. al, Evidence on COVID Mortality and Disparities Using Novel Measure, COVID Excess Mortality Percentage: Evidence from Indiana and Wisconsin (working paper 2023).

The underlying paper is available at <http://ssrn.com/abstract=4244039>

^1^ Corresponding author: Andy Ye Yuan, email: [andyyuan@law.northwestern.edu](mailto:andyyuan@law.northwestern.edu)

Contents

[Data and Methods: Additional Details 2](#_Toc144977328)

[Text Analysis to Determine Whether Death is Due to COVID-19 2](#_Toc144977329)

[Wisconsin and Indiana Population 2](#_Toc144977330)

[Measuring zip-SES 3](#_Toc144977331)

[CEMP as Predictor of COVID-19 Mortality for the Unvaccinated in 2020 3](#_Toc144977332)

[Actual versus Predicted Non-Covid Natural Mortality During Pandemic Period 4](#_Toc144977333)

[Using 2019 Natural Mortality as the CEMP Denominator 5](#_Toc144977334)

[Declining Hispanic Mortality Advantage 5](#_Toc144977335)

[Summary of Additional Results 6](#_Toc144977336)

[Summary of Tables 6](#_Toc144977337)

[Summary of Figures 7](#_Toc144977338)

[Additional References for Appendix, not Cited in the Text 7](#_Toc144977339)

[Table App-1. Comparison of COVID-19 deaths per text analysis, to ICD-10 codes 8](#_Toc144977340)

[Table App-2. Summary Statistics on Study Population 9](#_Toc144977341)

[Table App-3. Natural Deaths, COVID-19 Deaths, and Population by Age and Race/Ethnicity 11](#_Toc144977342)

[Table App-4a. Mortality Rates by Age and Race/Ethnicity for Wisconsin, Indiana, and Illinois (Female) 13](#_Toc144977343)

[Table App-4b. Mortality Rates by Age and Race/Ethnicity for Wisconsin, Indiana, and Illinois (Male) 15](#_Toc144977344)

[Table App-5. Confidence Intervals for the CEMP Ratios in text Table 2 17](#_Toc144977345)

[Table App-6. Multinomial Logit Model Separately for Men and Women 18](#_Toc144977346)

[Table App-7. State-Specific Results from Racial/Ethnic Disparities in COVID Mortality Rates: Multivariate Logit Analysis 19](#_Toc144977347)

[Table App-8. Alternate CEMP Rates and CEMP Ratios Using 2019 Natural Mortality in Denominator 21](#_Toc144977348)

[Table App-9. Comparison of CEMP and CEMP-2019 Rates and Ratios 23](#_Toc144977349)

[Table App-10. Comparison of CEMP Ratios for Minority Groups 25](#_Toc144977350)

[Table App-11. Correlations between COVID-19 Mortality Rate and Non-Covid Natural Mortality Rate within Racial/Ethnic Groups 27](#_Toc144977351)

[Figure App- 1. Correlation between Non-Covid Natural Mortality and COVID-19 Mortality: Counts 28](#_Toc144977352)

[Figure App-2. Correlation between 2019 Natural Mortality and 2020 COVID-19 Mortality 30](#_Toc144977353)

[Figure App-3. Actual versus Predicted Non-COVID Natural Mortality 32](#_Toc144977354)

[Figure App-4. Confidence Intervals for Actual vs. Predicted Non-Covid-NMRs 34](#_Toc144977355)

[Figure App-5. Evolution of Natural Mortality Rates for Hispanics versus Whites 36](#_Toc144977356)

# Data and Methods: Additional Details

## Text Analysis to Determine Whether Death is Due to COVID-19

We conduct text analysis of the cause of death fields in death certificates, described below, to determine which deaths are likely to be caused by COVID-19. Table App-1 compares the COVID-19 counts we determine using text analysis to those from the ICD-10 codes generated by the National Center for Health Statistics (NCHS). It is apparent that using ICD-10 codes produces many false negatives (deaths that we code as caused by COVID-19) and a smaller but still meaningful number of false positives (deaths that we code as caused by COVID-19). One source of false negatives is that for more recent deaths, especially in Indiana, the ICD-10 codes are missing. This is principally for more recent deaths and reflects the time needed for the certificates to be sent to NCHS, coded, and returned to Indiana and Wisconsin.

Most of the false negatives and false positives are not close cases. The NCHS coding is simply wrong. In Wisconsin, most of the false negatives involve deaths in Milwaukee County, which were coded as B99 (other and unspecified infectious diseases). We speculate that the coder at NCHS responsible for coding these death certificates did not realize there was a specific code for COVID-19 death.

The detailed code we used to identify deaths as probably due to COVID-19 is available from the authors on request. In brief, we counted as COVID-19 deaths those for which:

(i) COVID-19 or variants (such as SARS-2, SARS-COV-2, coronavirus) was listed as the principal cause of death (we view reliability for this category as high);

(ii) COVID-19 or variants were listed in the causal chain and the principal cause of death was likely to be caused by COVID-19 (for example, pneumonia, respiratory failure, hypoxia) (we view reliability for this category as high);

(iii) COVID-19 or variants were listed in the causal chain and the principal cause of death is one of the known potential outcomes of COVID-19 infection (for example, heart attack, stroke, sepsis) or involved underlying disease that was plausibly exacerbated by COVID-19 infection (for example, heart failure, COPD, dementia) (we view reliability for this category as moderate); and

(iv) we treated COVID-19 included in the “other significant conditions” field, but not in the causal chain fields, as part of the causal chain, if the principal cause of death was in group (ii) and there was no entry in the causal chain fields indicating another likely cause of death.

## Wisconsin and Indiana Population

We use natural, non-COVID deaths (the CEMP denominator) to address the potential for population health to differ systematically for different demographic groups. We also report population fatality rates that require estimates for the population of different groups. We use population estimates for 2020 by age, gender and race/ethnicity from the 2020 American Community Survey.^[[1]](#footnote-1)^ The mortality records have separate fields for race and for ethnicity (Hispanic or not). We use, as racial/ethnic categories, non-Hispanic White. Black, Non-Black Hispanic, and Asian and other. We lack sufficient sample size to further divide the Asian and other category when computing CEMP.

The ACS generally provides population within age ranges; a particular limitation is that it provides a single number for ages 85+. We assign the population within an overall ACS-based (age group)*gender*race/ethnicity group to individual years of age using survival probabilities by (year of age)*gender*race/ethnicity from NCHS. These probabilities are available for Whites, Blacks, and Hispanics. For Asian and other, we use overall population probabilities as the survival probabilities. We combine all persons aged 100+ (within gender*race/ethnicity) into a single 100+ group. To go from population counts by (year of age)*gender*race/ethnicity to counts by (age in months)*gender*race/ethnicity, we assign birth months randomly. This provides a starting population as of January 2020. We then roll this population forward a month at a time, subtracting decedents each month (to do so, we assign birth month randomly to the decedents). If an (age in months)*gender*race/ethnicity cell has decedents, but no people, as occasionally happens for higher ages and smaller racial/ethnic groups, we assign the decedent to the nearest cell in age-months with positive population. If there are more decedents in a cell than starting population (about 150 cases), we mechanically increase the starting population so that the ending population cannot be negative. We do not have data on inward or outward migration, so ignore how migration could affect population.

These manual adjustments are small relative to other sources of error in the population estimates. The largest sources are likely to be: (i) the ACS estimate of starting population (the ACS and Census estimates for 2020 differ by around 10,000 people);^[[2]](#footnote-2)^ and (ii) the ACS estimates of population percentages by race/ethnicity.

## Measuring zip-SES

We measure zip-SES in quartiles, using the Graham Social Deprivation Index, which we have found in prior work to be strongly predictive of COVID-19 mortality (Franchi et. al, 2023). This measure was developed by (Butler et al., 2013), is distributed by the Robert Graham Center, and is available at: <https://www.graham-center.org/rgc/maps-data-tools/sdi/social-deprivation-index.html>.

The quartiles are derived from the mortality data and contain roughly equal numbers of decedents for Indiana and Wisconsin combined.

# CEMP as Predictor of COVID-19 Mortality for the Unvaccinated in 2020

Figure App-1 shows the correlation in Indiana (Panel A), Wisconsin (Panel B), and Illinois (Panel C) between natural mortality in April-December 2019 (pre-COVID period) and COVID-19 mortality in April-December 2020 (COVID period, but pre-vaccine), for population groups defined by age (groups are 18-39, 40-49, 50-59, 60-69, 70-79, 80-89, and 90+, gender, race/ethnicity, and zip-SES (only Indiana, zip code is not available for Wisconsin). The Pearson correlation coefficients are 0.97 (Indiana), 0.99 (Wisconsin), and 0.93 (Illinois), consistent with non-COVID natural mortality rates strongly predicting COVID mortality rates for unvaccinated persons during the pre-vaccine period. See text Figure 1 for similar graphs with finer age groups for Non-COVID mortality in 2020 versus COVID-19 mortality in 2020, with finer age groups.

Further validation comes from the multivariate regression analysis in text Table 3, in which CEMP ratio estimates within groups defined solely by age are similar to multivariate estimates that adjust for other factors associated with COVID-19 mortality. This suggests that the CEMP measure already controls well for population health.

# Actual versus Predicted Non-Covid Natural Mortality During Pandemic Period

To the extent that our counts of COVID-19 deaths, although higher than NCHS counts, are still undercounts, this should show up in measures of excess non-COVID natural deaths, defined as non-COVID-19 natural deaths minus predicted levels based on extrapolation of mortality trends from the pre-pandemic period. Undercounting of COVID-19 deaths would lead to excess deaths generally being positive during the pandemic periods, and spiking during the periods when measured COVID-19 mortality spikes.

Figure App-2, Panel A reports monthly natural non-COVID-19 and all natural deaths for Indiana for 2017 – June 2022; Panels B and C are similar but for Wisconsin and Illinois. For the pandemic period, we also show predicted natural non-COVID deaths, based on linear extrapolation from 2017-2019 to the same calendar month during the pandemic period. Natural deaths (including COVID-19 deaths) show COVID-related peaks in late 2020 and late-2021-early 2022. In Indiana and Wisconsin, natural non-COVID-19 deaths do not have substantial corresponding spikes (beyond normal seasonal effects), which would be expected if COVID-19 deaths were undercounted. Predicted non-COVID natural deaths (dashed line) are close to measured deaths, sometimes higher or lower, including during the peak COVID-19 mortality periods, but with no obvious pattern. Thus, In both states, there is no evidence for a substantial number of excess non-COVID-19 natural deaths. These figures provide evidence that our text-based coding of COVID-19 deaths does a good job of capturing actual COVID-19 deaths, and thus provides a reasonably reliable CEMP numerator and denominator.

In Illinois, in contrast, there is evidence for some rise in non-COVID natural deaths during periods with high COVID-19 mortality. This suggests some undercounting of COVID-19 deaths, and therefore underestimates of CEMP. However, in unreported results, the undercounting is similar across racial/ethnic groups, and thus should not have an important effect on the minority/White CEMP or MR ratios reported in the text.

Another possibility, on which Figure App-2 also provide evidence, is that prior COVID-19 infection will lead to higher future deaths from natural causes, not directly linkable to the prior infection. Higher post-infection mortality would predict positive actual-minus-predicted non-COVID-19 natural mortality generally, not limited to periods of high COVID-19 mortality. There is evidence from other research of excess cardiovascular risk for a limited period of time following infection (Rezel-Potts et. al, 2022). However, the extent of excess deaths is not known. Any excess deaths for our sample are too small to be apparent from Figure App-2.

# Using 2019 Natural Mortality as the CEMP Denominator

In the text, we use contemporaneous non-COVID natural mortality as the denominator for the CEMP measure. A possible alternative would be to use natural mortality in 2019 as the denominator. This has both advantages and disadvantages relative to the measure we use. Given the high correlation between 2019 natural mortality and 2020 non-COVID natural mortality within age group*race/ethnicity buckets (see Figure App-2), we would expect to find similar results using either contemporaneous non-COVID natural mortality or 2019 natural mortality in the CEMP denominator. An advantage of using the 2019 denominator is that pre-Covid data are not affected by the COVID pandemic. In particular, bias in CEMP due to undercounting of COVID-19 deaths would no longer be a concern. A disadvantage is that the 2019 data do not reflect changes over time in population or in non-COVID sources of mortality. A further benefit of measuring CEMP using a contemporaneous denominator is that this approach permits the multivariate decomposition and measurement of confidence intervals in Table 3.

We provide evidence below for a secular decline in the Hispanic life expectancy advantage. That decline implies that using a denominator based on 2019 natural mortality will cause upward bias in CEMP for Hispanics, relative to a contemporaneous denominator by suppressing the denominator.

Nonetheless, using 2019 natural mortality in the CEMP denominator is a useful robustness check on our results. Table App-8 reproduces text Table 2 but uses natural mortality in 2019 as the denominator – we call this measure CEMP-2019. CEMP-2019 levels are typically a bit higher than CEMP levels, consistent with the modest undercounting of COVID deaths that we find in Illinois (Figure App-3), so these differences are expected. Table App-9 directly compares minority/White CEMP ratios from the two approaches. These ratios are similar with both denominators.

# Declining Hispanic Mortality Advantage

Figure App-5 responds to a referee suggestion that the Hispanic life expectancy advantage over Whites might be eroding over time. If so, the factors that underlie convergence in life expectancy might in the near term manifest more strongly in COVID-19 risk, such as obesity and diabetes, than in mortality from other natural causes. This could explain at least part of the elevated COVID-19 mortality rates we find for Hispanics.

We were able to assess the possibility that the Hispanic mortality advantage was declining over time with a dataset, from the National Center for Health Statistics, that provides individual death certificates, including race/ethnicity, with geography limited to the county level and with a longer delay before results are available than the three-state data we rely on. In Figure App-5, we show relative national mortality rates by age group for Hispanics and for non-Hispanic Whites in selected years from 2010 through 2020 (for 2020, we use non-COVID natural mortality).

The Hispanic/White mortality rate ratios are consistently well below 1.00 in all years. This is the Hispanic paradox, to which COVID-19 mortality was an exception. However, the Hispanic advantage in natural mortality is slowly but steadily declining over time – the ratios are rising toward 1.00. The change in mortality rates is slow, so should not strongly affect our results.

The decline in the Hispanic advantage, by itself, would imply that Hispanic CEMP levels, which use 2020 non-COVID natural mortality rates in the denominator, should be lower than Hispanic/White CEMP-2019 levels, which use 2019 natural mortality rates in the denominator. We observe this difference in Table App-10. However, as Table App-10 also shows, Hispanic/White CEMP ratios are similar to CEMP-2019 ratios, suggesting that other differences between 2019 and 2020 non-COVID mortality rates offset any effects from the decline in magnitude of the Hispanic life expectancy advantage. However, this does not directly affect the referee’s speculation that the factors that drive convergence in life expectancy might affect COVID-19 risk more strongly than mortality from other natural causes.

# Summary of Additional Results

## Summary of Tables

Table App-1 is discussed above. It shows the differences between COVID-19 deaths counted using our text-based measure, and deaths counted using ICD-10 codes determined from the text fields in death certificates.

Table App-2 is similar to Table 1 in text, but provides summary statistics on the study population separately for Wisconsin, Indiana, and Illinois.

Table App-3 provides information on COVID-19 deaths, other natural deaths, and population estimates by time period for Indiana, Wisconsin, and Illinois taken together. These data are used to calculate the CEMP, MR, and CEMP Ratios to White in text Table 2.

Tables App-4a (for women) and 4b (for men), are similar to text Table 2 but provide separate results for women and men.

Table App-5 provides 95% confidence intervals for the CEMP ratios to White reported in text Table 2. The CIs are based on logistic regression, as described in the text, but without covariates for zip-SES, education, or marital status.

Table App-6 provides multivariate logistic results similar to text Table 3, but separately for women (Panel A) and men (Panel B).

Table App-7 provides state-specific versions of the multinomial logit regressions. These can be used to assess to what extent the results we report vary across the three states on our sample.

Table App-8 is similar to text Table 2, but reports results for CEMP-2019 (the denominator is natural mortality in 2019) instead of CEMP. See discussion above of the CEMP-2019 measure.

Table App-9 compares CEMP levels and minority/White CEMP ratios, to the corresponding CEMP-2019 levels and minority/White ratios.

Table App-10 Table supplements text Table 2 by providing an expanded set of CEMP ratios for different race/ethnic groups. The additional ratios included in this table are Hispanic/Black; Other/Black, and Other/Hispanic.

Table App-10 Table supplements text Figure 1, which reports correlations between COVID-MR and Non-Covid NMR for the full sample. The table provides Pearson correlation coefficients within each racial/ethnic group.

## Summary of Figures

Text Figure 1 provides scatterplots of Covid-MR mortality *rates* versus Non-Covid natural mortality *rates* for each of our time periods, expressed as deaths per 100 thousand population. The text figure shows data points for groups defined by state * age (18-39, 40-49, 50-59, 60-64, 65-69, 70-74, 75-79, 80-84, 85-89, 90-94, 95+) * gender * race/ethnicity (168 groups). Figure App-1 presents alternative scatterplots of of the number of Covid-19 *deaths* versus non-Covid natural *deaths* for groups by state * age (18-39, 40-49, 50-59, 60-64, 65-69, 70-74, 75-79, 80-84, 85-89, 90-94, 95+) * gender * race/ethnicity (840 groups). Switching from rates to counts lets us use finer age groups. We cannot condition on zip-SES in the text because the American Community Survey does not provide population estimates divided this finely.. Note that the y-axis runs from 0-350 in the pre-vaccine period, versus from 0-150 in the early vaccine period).

Figure App-2 provides a scatterplot and best fit regression line comparing 2019 natural mortality and 2020 non-COVID natural mortality.

Figures App-3 and App-4 provide evidence the extent to which COVID-19 mortality was undercounted (implying downward bias in CEMP in Illinois, Indiana, and Wisconsin. Figure App-3 provides, separately for Illinois, Indiana, and Wisconsin, three lines, for all natural deaths (red line, affected by COVID-19), measured natural-non-COVID deaths from March 2020 on (blue line) , and predicted non-COVID deaths beginning in March 2020, based on linear extrapolation of mortality for the same month in the pre-COVID period (dashed green line). Undercounting of COVID-19 deaths would mean overcounting of natural non-Covid deaths, which would imply that the measured line should be above the predicted line, especially in periods with a high number of COVID deaths.

Figure App-4 drops the line for all natural deaths and adds 95% confidence intervals (CIs) around predicted non-Covid natural deaths.

For Indiana and Wisconsin, there is no evidence of important undercounting. The measured line for non-Covid natural deaths is generally within the 95% CE around the predicted line, includig during peak COVID-19 periods. For Illinois, in contrast, we find evidence of some undercounting of COVID-19 deaths. The measured blue line is generally above both the predicted line and is often above the upper end of the the 95% CI, especially in the first few pandemic months.

Figure App-5 is discussed above. It provides evidence on the gradual erosion of the Hispanic advantage in non-COVID natural mortality.

# Additional References for Appendix, not Cited in the Text

Franchi, Lorenzo, Natalia Barreto, Anna Chorniy, John Meurer, and Bernard Black, A Comparative Assessment of Measures of Area-Level Socio-Economic Status (working paper 2023), at <http://ssrn.com/abstract=4030029>.

Rezel-Potts, Emma, Abdel Douiri, Xiaohui Sun, Phillip J. Chowienczyk, Ajay M. Shah, and Martin C. Gulliford (2022), Cardiometabolic outcomes up to 12 months after COVID-19 infection. A matched cohort study in the UK, *PLoS*, <https://doi.org/10.1371/journal.pmed.1004052>.

# Table App-1. Comparison of COVID-19 deaths per text analysis, to ICD-10 codes

Table shows counts, for April 2020 through June 2022 (for Illinois through December 2021), of COVID-19 deaths determined from our text analysis to COVID-19 deaths, determined using ICD-10 codes (the code for COVID-19 cause of death is U07.1) ICD-10 codes are generated by NCHS, based on the text fields in the death certificates.

| **Wisconsin (thru June 2022)** | COVID-19 by Text Analysis | non-COVID-19 by Text Analysis | **Total** |
| --- | --- | --- | --- |
| COVID-19 by ICD Code | 10,931 | 206 | **11,137** |
| non-COVID-19 by ICD Code | 1,458 | 109,407 | **110,865** |
| Missing ICD Code (mostly recent cases) | 3 | 77 | **80** |
| **Total** | **12,392** | **109,690** | **122,082** |
| From Natural Causes |  |  | 109,872 |
| **Indiana (thru June 2022)** |  |  |  |
| COVID-19 by ICD Code | 18,156 | 322 | **11,137** |
| non-COVID-19 by ICD Code | 1,116 | 126,837 | **110,865** |
| Missing ICD Code (mostly recent cases) | 1,192 | 6,929 | **80** |
| **Total** | **20,464** | **134,088** | **122,082** |
| From Natural Causes |  |  | 142,575 |
| **Illinois (thru December 2021)** |  |  |  |
| COVID-19 by ICD-10 Code | 26,928 | 401 | **27,329** |
| non-COVID-19 by ICD-10 Code | 739 | 203,500 | **204,239** |
| Missing ICD-10 Code | 23 | 308 | **331** |
| **Total** | **27,690** | **204,209** | **231,899** |
| From Natural Causes |  |  | 212,795 |

# Table App-2. Summary Statistics on Study Population

Table is similar to text Table 1, but shows summary statistics separately for Wisconsin, Indiana, and Illinois. Deaths are for April 2020 through June 2022 for Indiana and Wisconsin, and through December 2021 for Illinois.

|  | **Wisconsin (age 18+) thru June 2022** | | | **Indiana (age 18+) thru June 2022** | | | **Illinois (age 18+) thru December 2021** | | |
| --- | --- | --- | --- | --- | --- | --- | --- | --- | --- |
|  | **Covid Deaths**  **N (%)** | **Non-Covid Natural Deaths**  **N (%)** | **Total Population**  **N (%)** | **Covid Deaths**  **N (%)** | **Non-Covid Natural Deaths N (%)** | **Total Population**  **N (%)** | **Covid Deaths**  **N (%)** | **Non-Covid Natural Deaths N (%)** | **Total Population**  **N (%)** |
| ***Female*** | 5,635  (44.8%) | 54,308  (49.6%) | 2,295,165  (50.6%) | 9,901  (46.6%) | 69,577  (49.9%) | 2,629,666  (51.3%) | 12,062  (44.4%) | 91,238  (50.5%) | 5,070,631  (51.4%) |
| ***Male*** | 6,941  (55.2%) | 55,162  (50.4%) | 2,236,842  (49.4%) | 11,347  (53.4%) | 69,721  (50.1%) | 2,495,676  (48.7%) | 15,093  (55.6%) | 89,465  (49.5%) | 4,789,261  (48.6%) |
| ***Race/Ethnicity*** |  |  |  |  |  |  |  |  |  |
| *White* | 10,743  (85.42%) | 99,567  (90.95%) | 3,796,370  (83.77%) | 18,181  (85.57%) | 124,127  (89.11%) | 4,150,952  (80.99%) | 17,168  (63.22%) | 136,773  (75.69%) | 6,287,972  (63.77%) |
| *Hispanic* | 588  (4.68%) | 1,833  (1.67%) | 250,089  (5.52%) | 840  (3.95%) | 2,437  (1.75%) | 297,624  (5.81%) | 4,110  (15.14%) | 9,654  (5.34%) | 1,490,756  (15.12%) |
| *Black* | 843  (6.70%) | 5,889  (5.38%) | 256,029  (5.62%) | 2,007  (9.45%) | 11,599  (8.33%) | 456,194  (8.90%) | 4,887  (18.00%) | 29,660  (16.41%) | 1,358,035  (13.77%) |
| *Other (incl. Asian)* | 402  (3.20%) | 2,181  (1.99%) | 229,519  (5.06%) | 220  (1.04%) | 1,135  (0.81%) | 220,572  (4.30%) | 990  (3.65%) | 4,616  (2.55%) | 723,129  (7.34%) |
| ***Age*** |  |  |  |  |  |  |  |  |  |
| *18-39* | 221  (1.8%) | 1,790  (1.6%) | 1,641,004  (36.2%) | 339  (1.6%) | 2,766  (2.0%) | 1,951,077  (38.1%) | 554  (2.0%) | 3,296  (1.8%) | 3,761,377  (38.2%) |
| *40-49* | 395  (3.1%) | 2,628  (2.4%) | 724,030  (16.0%) | 723  (3.4%) | 4,471  (3.2%) | 834,248  (16.3%) | 1,034  (3.8%) | 5,374  (3.0%) | 1,641,498  (16.7%) |
| *50-59* | 1,017  (8.1%) | 7,828  (7.2%) | 784,143  (17.3%) | 1,769  (8.3%) | 11,946  (8.6%) | 862,458  (16.3%) | 2,385  (8.8%) | 14,567  (8.1%) | 1,657,266  (16.8%) |
| *60-69* | 2,090  (16.6%) | 18,028  (16.5%) | 699,990  (15.4%) | 3,912  (18.4%) | 25,916  (18.6%) | 748,688  (14.6%) | 4,953  (18.2%) | 31,777  (17.6%) | 1,408,848  (14.3%) |
| *70-79* | 3,087  (24.5%) | 25,948  (23.7%) | 427,506  (9.4%) | 5,574  (26.2%) | 35,063  (25.2%) | 460,140  (9.0%) | 6,919  (25.5%)) | 42,796  (23.7%) | 866,099  (8.8%) |
| *80-89* | 3,462  (27.5%) | 30,989  (28.3%) | 201,869  (4.5%) | 5,718  (26.9%) | 36,332  (26.1%) | 213,652  (4.2%) | 6,997  (25.8%) | 48,459  (26.8%) | 414,829  (4.2%) |
| *90+* | 2,304  (18.3%) | 22,255  (20.3%) | 53,465  (1.2%) | 3,213  (15.1%) | 22,804  (16.4%) | 55,079  (1.1%) | 4,313  (15.9%) | 34,425  (19.1%) | 109,975  (1.1%) |
| ***Zip-SES (1 = highest)*** | |  |  |  |  |  |  |  |  |
| *Quartile 1* | 3,547  (28.2%) | 32,216  (29.4%) | 1,114,406  (24.6%) | 5,097  (24.0%) | 36,199  (26.0%) | 862,590  (16.9%) | 5,750  (21.2%) | 48,618  (26.9%) | 2,132,755  (21.6%) |
| *Quartile 2* | 3,515  (28.0%) | 31,215  (28.5%) | 1,260,705  (27.9%) | 5,331  (25.1%) | 34,138  (24.5%) | 1,009,005  (19.7%) | 6,621  (24.4%) | 45,808  (25.4%) | 2,022,231  (20.5%) |
| *Quartile 3* | 2,837  (22.6%) | 25,854  (23.6%) | 1,496,515  (33.1%) | 5,690  (26.8%) | 35,961  (25.8%) | 1,722,387  (33.7%) | 6,991  (25.8%) | 44,732  (24.8%) | 2,448,129  (24.8%) |
| *Quartile 4* | 2,632  (20.9%) | 19,927  (18.2%) | 653,930  (14.4%) | 5,058  (23.8%) | 32,548  (23.4%) | 1,517,590  (29.7%) | 7,774  (28.6%) | 41,446  (22.9%) | 3,251,360  (33.0%) |
| ***Education*** |  |  |  |  |  |  |  |  |  |
| *Unknown* | 210  (1.7%) | 1,566  (1.4%) |  | 293  (1.4%) | 1,556  (1.1%) |  | 604  (2.2%) | 3,116  (1.7%) | n.a. |
| *Not high school grad* | 2,018  (16.0%) | 15,182  (13.9%) | 295,211  (7.4%) | 3,807  (17.9%) | 23,344  (16.8%) | 476,425  (10.7%) | 5,729  (21.1%) | 27,485  (15.2%) | 898,338  (10.3%) |
| *High school grad* | 6,249  (49.7%) | 53,860  (49.2%) | 1,205,327  (30.3%) | 10,841  (51.0%) | 71,888  (51.6%) | 1,482,376  (33.2%) | 12,136  (44.7%) | 83,740  (46.3%) | 2,220,385  (25.6%) |
| *Some college* | 2,343  (18.6%) | 21,042  (19.2%) | 1,255,058  (31.5%) | 3,645  (17.2%) | 23,549  (16.9%) | 1,294,562  (29.0%) | 4,960  (18.3%) | 34,589  (19.1%) | 2,482,663  (28.6%) |
| *College grad or higher* | 1,756  (14.0%) | 17,820  (16.3%) | 1,226,567  (30.8%) | 2,662  (12.5%) | 18,961  (13.6%) | 1,212,778  (27.2%) | 3,726  (13.7%) | 31,773  (17.6%) | 3,085,273  (35.5%) |

# Table App-3. Natural Deaths, COVID-19 Deaths, and Population by Age and Race/Ethnicity

COVID-19 deaths, non-COVID natural deaths, all natural deaths, and population for Indiana, Wisconsin, and Illinois (based on ACS data for 2020) during designated periods. Table shows separate results for non-Hispanic White, Black, non-Black Hispanic, and Asian-and-other. Total adult population is 19,519,265 persons, adult COVID-19 deaths are 61,1381; and adult non- COVID-19 natural deaths are 427,98. Indiana and Wisconsin data is available through June 30, 2022; Illinois is available through December 31, 2021.

**Panel A. Pre-Vaccine Period (April-December 2020). Indiana, Wisconsin, and Illinois**

|  | **White** | | | **Black** | | | **Hispanic** | | | **Asian-and other** | | |
| --- | --- | --- | --- | --- | --- | --- | --- | --- | --- | --- | --- | --- |
| Age | Covid Deaths | Non-Covid Natural Deaths | Population | Covid Deaths | Non-Covid Natural Deaths | Population | Covid Deaths | Non-Covid Natural Deaths | Population | Covid Deaths | Non-Covid Natural Deaths | Population |
| 18-39 | 77 | 1,580 | 4,803,194 | 103 | 700 | 906,958 | 132 | 261 | 1,061,329 | 22 | 105 | 581,977 |
| 40-49 | 204 | 2,923 | 2,245,762 | 176 | 1,114 | 346,175 | 271 | 432 | 403,825 | 31 | 124 | 204,014 |
| 50-59 | 743 | 9,762 | 2,520,180 | 442 | 2,472 | 334,909 | 569 | 722 | 287,139 | 71 | 295 | 161,639 |
| 60-69 | 2,583 | 21,908 | 2,304,749 | 991 | 4,843 | 262,104 | 841 | 1,104 | 168,273 | 182 | 578 | 122,400 |
| 70-79 | 5,439 | 31,875 | 1,457,774 | 1,148 | 4,440 | 144,450 | 764 | 1,141 | 79,544 | 267 | 761 | 71,977 |
| 80-89 | 7,494 | 38,360 | 712,819 | 931 | 3,778 | 60,492 | 539 | 1,208 | 30,752 | 246 | 806 | 26,287 |
| 90+ | 5,422 | 28,245 | 190,816 | 442 | 1,872 | 15,170 | 220 | 653 | 7,607 | 133 | 469 | 4,926 |
| 18-59 | 1,024 | 14,265 | 9,569,136 | 721 | 4,286 | 1,588,042 | 972 | 1,415 | 1,752,293 | 124 | 524 | 947,630 |
| 60+ | 20,938 | 120,388 | 4,666,158 | 3,512 | 14,933 | 482,216 | 2,364 | 4,106 | 286,176 | 828 | 2,614 | 225,590 |
| Total | 21,962 | 134,653 | 14,235,294 | 4,233 | 19,219 | 2,070,258 | 3,336 | 5,521 | 2,038,469 | 952 | 3,138 | 1,173,220 |

**Panel B. Partial-Vaccine Period (January-June 2021). Indiana, Wisconsin, and Illinois**

|  | **White** | | | **Black** | | | **Hispanic** | | | **Asian-and other** | | |
| --- | --- | --- | --- | --- | --- | --- | --- | --- | --- | --- | --- | --- |
| Age | Covid Deaths | Non-Covid Natural Deaths | Population | Covid Deaths | Non-Covid Natural Deaths | Population | Covid Deaths | Non-Covid Natural Deaths | Population | Covid Deaths | Non-Covid Natural Deaths | Population |
| 18-39 | 62 | 1,059 | 4,803,194 | 50 | 435 | 906,958 | 43 | 181 | 1,061,329 | 6 | 63 | 581,977 |
| 40-49 | 137 | 1,954 | 2,245,762 | 91 | 668 | 346,175 | 105 | 263 | 403,825 | 14 | 76 | 204,014 |
| 50-59 | 498 | 6,121 | 2,520,180 | 202 | 1,597 | 334,909 | 191 | 485 | 287,139 | 39 | 200 | 161,639 |
| 60-69 | 1,243 | 14,355 | 2,304,749 | 355 | 3,006 | 262,104 | 298 | 726 | 168,273 | 64 | 361 | 122,400 |
| 70-79 | 2,179 | 21,279 | 1,457,774 | 366 | 2,876 | 144,450 | 260 | 797 | 79,544 | 97 | 464 | 71,977 |
| 80-89 | 2,259 | 24,237 | 712,819 | 258 | 2,270 | 60,492 | 151 | 779 | 30,752 | 64 | 544 | 26,287 |
| 90+ | 1,352 | 16,781 | 190,816 | 111 | 1,172 | 15,170 | 54 | 421 | 7,607 | 31 | 280 | 4,926 |
| 18-59 | 697 | 9,134 | 9,569,136 | 343 | 2,700 | 1,588,042 | 339 | 929 | 1,752,293 | 59 | 339 | 947,630 |
| 60+ | 7,033 | 76,652 | 4,666,158 | 1,090 | 9,324 | 482,216 | 763 | 2,723 | 286,176 | 256 | 1,649 | 225,590 |
| Total | 7,730 | 85,786 | 14,235,294 | 1,433 | 12,024 | 2,070,258 | 1,102 | 3,652 | 2,038,469 | 315 | 1,988 | 1,173,220 |

**Panel C. Delta Period (July-December 2021). Indiana, Wisconsin, and Illinois**

|  | **White** | | | **Black** | | | **Hispanic** | | | **Asian-and other** | | |
| --- | --- | --- | --- | --- | --- | --- | --- | --- | --- | --- | --- | --- |
| Age | Covid Deaths | Non-Covid Natural Deaths | Population | Covid Deaths | Non-Covid Natural Deaths | Population | Covid Deaths | Non-Covid Natural Deaths | Population | Covid Deaths | Non-Covid Natural Deaths | Population |
| 18-39 | 279 | 1,073 | 4,803,194 | 129 | 496 | 906,958 | 89 | 189 | 1,061,329 | 24 | 77 | 581,977 |
| 40-49 | 590 | 2,081 | 2,245,762 | 164 | 728 | 346,175 | 144 | 289 | 403,825 | 24 | 86 | 204,014 |
| 50-59 | 1,440 | 6,414 | 2,520,180 | 288 | 1,635 | 334,909 | 172 | 481 | 287,139 | 28 | 201 | 161,639 |
| 60-69 | 2,651 | 15,196 | 2,304,749 | 432 | 3,176 | 262,104 | 204 | 742 | 168,273 | 51 | 347 | 122,400 |
| 70-79 | 3,086 | 22,816 | 1,457,774 | 343 | 2,841 | 144,450 | 148 | 803 | 79,544 | 66 | 481 | 71,977 |
| 80-89 | 2,614 | 25,853 | 712,819 | 221 | 2,382 | 60,492 | 101 | 790 | 30,752 | 42 | 538 | 26,287 |
| 90+ | 1,242 | 18,178 | 190,816 | 90 | 1,182 | 15,170 | 42 | 454 | 7,607 | 19 | 282 | 4,926 |
| 18-59 | 2,309 | 9,568 | 9,569,136 | 581 | 2,859 | 1,588,042 | 405 | 959 | 1,752,293 | 76 | 364 | 947,630 |
| 60+ | 9,593 | 82,043 | 4,666,158 | 1,086 | 9,581 | 482,216 | 495 | 2,789 | 286,176 | 178 | 1,648 | 225,590 |
| Total | 11,902 | 91,611 | 14,235,294 | 1,667 | 12,440 | 2,070,258 | 900 | 3,748 | 2,038,469 | 254 | 2,012 | 1,173,220 |

**Panel D. Omicron Period (January-June 2022). Indiana and Wisconsin**

|  | **White** | | | **Black** | | | **Hispanic** | | | **Asian-and other** | | |
| --- | --- | --- | --- | --- | --- | --- | --- | --- | --- | --- | --- | --- |
| Age | Covid Deaths | Non-Covid Natural Deaths | Population | Covid Deaths | Non-Covid Natural Deaths | Population | Covid Deaths | Non-Covid Natural Deaths | Population | Covid Deaths | Non-Covid Natural Deaths | Population |
| 18-39 | 79 | 585 | 2,711,886 | 14 | 140 | 332,268 | 16 | 56 | 301,159 | 7 | 27 | 246,768 |
| 40-49 | 139 | 1,071 | 1,253,412 | 33 | 220 | 122,272 | 26 | 96 | 108,884 | 11 | 53 | 73,710 |
| 50-59 | 455 | 3,199 | 1,406,015 | 52 | 467 | 111,434 | 22 | 124 | 72,110 | 17 | 123 | 57,042 |
| 60-69 | 934 | 8,406 | 1,283,943 | 137 | 888 | 82,924 | 47 | 182 | 40,212 | 21 | 162 | 41,599 |
| 70-79 | 1,330 | 12,513 | 805,828 | 103 | 888 | 42,186 | 52 | 189 | 17,202 | 28 | 186 | 22,430 |
| 80-89 | 1,194 | 13,734 | 385,002 | 84 | 626 | 16,723 | 31 | 197 | 6,492 | 13 | 186 | 7,304 |
| 90+ | 638 | 9,331 | 101,236 | 38 | 296 | 4,416 | 13 | 97 | 1,654 | 5 | 84 | 1,238 |
| 18-59 | 673 | 4,855 | 5,371,313 | 99 | 827 | 565,974 | 64 | 276 | 482,153 | 35 | 203 | 377,520 |
| 60+ | 4,096 | 43,984 | 2,576,009 | 362 | 2,698 | 146,249 | 143 | 665 | 65,560 | 67 | 618 | 72,571 |
| Total | 4,769 | 48,839 | 7,947,322 | 461 | 3,525 | 712,223 | 207 | 941 | 547,713 | 102 | 821 | 450,091 |

# Table App-4a. Mortality Rates by Age and Race/Ethnicity for Wisconsin, Indiana, and Illinois (Female)

COVID-19 MR, Non-COVID natural mortality rate, and CEMP for Wisconsin, Indiana, and Illinois females during indicated time periods. The Illinois data ends December 31, 2021. The last column in each panel for Black, Hispanic, and Other reports the ratio of CEMP to the corresponding CEMP for White. See Table App-4 for confidence intervals for all reported CEMP Ratio to White values. All panels. Data is for decedents so results are effectively weighted by mortality, not population. *, **, *** indicates p < .05, .01, and .001, respectively; significant results (at p < .05 or better) in boldface.

MR **=** $\frac{COVID deaths}{Population}$ ; Non-COVID Natural Mortality Rate (NMR) =$\frac{Non-Covid natural deaths}{Population}$ ; CEMP **=** $\frac{COVID deaths}{Non-COVID natural deaths}$

**Panel A. Pre-Vaccine Period (April-December 2020). Indiana, Wisconsin, and Illinois**

|  | **White** | | | | **Black** | | | | **Hispanic** | | | | **Other** | | | |
| --- | --- | --- | --- | --- | --- | --- | --- | --- | --- | --- | --- | --- | --- | --- | --- | --- |
| Age | COVID MR | Non-Covid NMR | CEMP |  | COVID MR | Non-Covid NMR | CEMP | CEMP Ratio to White | COVID MR | Non-Covid NMR | CEMP | CEMP Ratio to White | COVID MR | Non-Covid NMR | CEMP | CEMP Ratio to White |
| 18-39 | 0.001% | 0.028% | 4.93% |  | 0.010% | 0.068% | 14.29% | 2.90 | 0.007% | 0.018% | 39.13% | 7.94 | 0.003% | 0.018% | 17.65% | 3.58 |
| 40-49 | 0.006% | 0.109% | 5.67% |  | 0.039% | 0.255% | 15.29% | 2.70 | 0.031% | 0.074% | 42.25% | 7.46 | 0.010% | 0.039% | 26.83% | 4.74 |
| 50-59 | 0.022% | 0.305% | 7.35% |  | 0.105% | 0.595% | 17.63% | 2.40 | 0.112% | 0.162% | 69.51% | 9.46 | 0.032% | 0.124% | 25.47% | 3.47 |
| 60-69 | 0.080% | 0.742% | 10.79% |  | 0.273% | 1.429% | 19.08% | 1.77 | 0.297% | 0.519% | 57.24% | 5.31 | 0.086% | 0.378% | 22.83% | 2.12 |
| 70-79 | 0.279% | 1.838% | 15.18% |  | 0.617% | 2.510% | 24.57% | 1.62 | 0.645% | 1.224% | 52.71% | 3.47 | 0.261% | 0.885% | 29.53% | 1.95 |
| 80-89 | 0.867% | 4.766% | 18.20% |  | 1.249% | 5.709% | 21.88% | 1.20 | 1.346% | 3.607% | 37.33% | 2.05 | 0.797% | 2.809% | 28.37% | 1.56 |
| 90+ | 2.595% | 14.339% | 18.10% |  | 2.656% | 11.929% | 22.26% | 1.23 | 2.161% | 7.670% | 28.17% | 1.56 | 2.653% | 9.091% | 29.18% | 1.61 |
| 18-59 | 0.008% | 0.121% | 6.71% |  | 0.037% | 0.225% | 16.47% | 2.45 | 0.030% | 0.054% | 54.92% | 8.18 | 0.010% | 0.041% | 23.74% | 3.54 |
| 60+ | 0.406% | 2.470% | 16.43% |  | 0.606% | 2.761% | 21.94% | 1.34 | 0.585% | 1.335% | 43.86% | 2.67 | 0.297% | 1.067% | 27.82% | 1.69 |
| Total | 0.146% | 0.937% | 15.61% |  | 0.182% | 0.870% | 20.88% | 1.34 | 0.113% | 0.247% | 45.93% | 2.94 | 0.069% | 0.254% | 27.30% | 1.75 |

**Panel B. Early Vaccine Period (January-June 2021). Indiana, Wisconsin, and Illinois**

|  | **White** | | | | **Black** | | | | **Hispanic** | | | | **Other** | | | |
| --- | --- | --- | --- | --- | --- | --- | --- | --- | --- | --- | --- | --- | --- | --- | --- | --- |
| Age | COVID MR | Non-Covid NMR | CEMP |  | COVID MR | Non-Covid NMR | CEMP | CEMP Ratio to White | COVID MR | Non-Covid NMR | CEMP | CEMP Ratio to White | COVID MR | Non-Covid NMR | CEMP | CEMP Ratio to White |
| 18-39 | 0.001% | 0.018% | 6.00% |  | 0.007% | 0.040% | 17.39% | 2.90 | 0.002% | 0.012% | 15.00% | 2.50 | 0.000% | 0.010% | 0.00% | 0.00 |
| 40-49 | 0.004% | 0.070% | 5.61% |  | 0.027% | 0.157% | 17.24% | 3.08 | 0.012% | 0.048% | 25.81% | 4.60 | 0.008% | 0.030% | 25.00% | 4.46 |
| 50-59 | 0.015% | 0.192% | 7.67% |  | 0.043% | 0.383% | 11.24% | 1.47 | 0.040% | 0.109% | 36.42% | 4.75 | 0.016% | 0.088% | 18.67% | 2.43 |
| 60-69 | 0.038% | 0.483% | 7.91% |  | 0.105% | 0.924% | 11.36% | 1.44 | 0.104% | 0.338% | 30.74% | 3.89 | 0.037% | 0.226% | 16.45% | 2.08 |
| 70-79 | 0.108% | 1.232% | 8.76% |  | 0.224% | 1.619% | 13.85% | 1.58 | 0.225% | 0.849% | 26.54% | 3.03 | 0.076% | 0.574% | 13.30% | 1.52 |
| 80-89 | 0.246% | 2.977% | 8.26% |  | 0.358% | 3.444% | 10.41% | 1.26 | 0.370% | 2.333% | 15.87% | 1.92 | 0.137% | 1.953% | 7.02% | 0.85 |
| 90+ | 0.623% | 8.505% | 7.33% |  | 0.603% | 7.581% | 7.96% | 1.09 | 0.506% | 5.159% | 9.81% | 1.34 | 0.507% | 6.110% | 8.29% | 1.13 |
| 18-59 | 0.005% | 0.077% | 7.03% |  | 0.019% | 0.141% | 13.70% | 1.95 | 0.010% | 0.036% | 28.95% | 4.12 | 0.005% | 0.028% | 16.06% | 2.28 |
| 60+ | 0.125% | 1.554% | 8.07% |  | 0.196% | 1.745% | 11.22% | 1.39 | 0.184% | 0.888% | 20.73% | 2.57 | 0.074% | 0.703% | 10.57% | 1.31 |
| Total | 0.047% | 0.590% | 7.98% |  | 0.064% | 0.549% | 11.69% | 1.47 | 0.037% | 0.164% | 22.26% | 2.79 | 0.019% | 0.169% | 11.31% | 1.42 |

**Panel C. Delta Period (July-December 2021). Indiana, Wisconsin, and Illinois**

|  | **White** | | | | **Black** | | | | **Hispanic** | | | | **Other** | | | |
| --- | --- | --- | --- | --- | --- | --- | --- | --- | --- | --- | --- | --- | --- | --- | --- | --- |
| Age | COVID MR | Non-Covid NMR | CEMP |  | COVID MR | Non-Covid NMR | CEMP | CEMP Ratio to White | COVID MR | Non-Covid NMR | CEMP | CEMP Ratio to White | COVID MR | Non-Covid NMR | CEMP | CEMP Ratio to White |
| 18-39 | 0.005% | 0.019% | 26.46% |  | 0.013% | 0.052% | 25.21% | 0.95 | 0.003% | 0.012% | 27.87% | 1.05 | 0.002% | 0.009% | 28.00% | 1.06 |
| 40-49 | 0.019% | 0.079% | 23.86% |  | 0.042% | 0.184% | 22.94% | 0.96 | 0.014% | 0.049% | 28.42% | 1.19 | 0.008% | 0.029% | 25.81% | 1.08 |
| 50-59 | 0.042% | 0.199% | 21.20% |  | 0.068% | 0.386% | 17.74% | 0.84 | 0.031% | 0.124% | 25.15% | 1.19 | 0.014% | 0.081% | 17.39% | 0.82 |
| 60-69 | 0.089% | 0.522% | 17.08% |  | 0.150% | 0.915% | 16.38% | 0.96 | 0.093% | 0.313% | 29.77% | 1.74 | 0.039% | 0.205% | 18.84% | 1.10 |
| 70-79 | 0.167% | 1.328% | 12.58% |  | 0.235% | 1.654% | 14.19% | 1.13 | 0.140% | 0.921% | 15.21% | 1.21 | 0.052% | 0.547% | 9.46% | 0.75 |
| 80-89 | 0.280% | 3.190% | 8.77% |  | 0.333% | 3.587% | 9.28% | 1.06 | 0.213% | 2.446% | 8.72% | 0.99 | 0.098% | 1.901% | 5.15% | 0.59 |
| 90+ | 0.559% | 9.043% | 6.18% |  | 0.531% | 7.464% | 7.12% | 1.15 | 0.487% | 5.490% | 8.87% | 1.43 | 0.387% | 5.544% | 6.99% | 1.13 |
| 18-59 | 0.018% | 0.081% | 22.42% |  | 0.032% | 0.155% | 20.53% | 0.92 | 0.010% | 0.039% | 26.61% | 1.19 | 0.006% | 0.026% | 21.60% | 0.96 |
| 60+ | 0.170% | 1.667% | 10.20% |  | 0.216% | 1.766% | 12.23% | 1.20 | 0.134% | 0.919% | 14.62% | 1.43 | 0.059% | 0.662% | 8.96% | 0.88 |
| Total | 0.071% | 0.632% | 11.23% |  | 0.079% | 0.565% | 13.92% | 1.24 | 0.029% | 0.171% | 16.93% | 1.51 | 0.017% | 0.158% | 10.60% | 0.94 |

**Panel D. Omicron Period (January-June 2022). Indiana and Wisconsin**

|  | **White** | | | | **Black** | | | | **Hispanic** | | | | **Other** | | | |
| --- | --- | --- | --- | --- | --- | --- | --- | --- | --- | --- | --- | --- | --- | --- | --- | --- |
| Age | COVID MR | Non-Covid NMR | CEMP |  | COVID MR | Non-Covid NMR | CEMP | CEMP Ratio to White | COVID MR | Non-Covid NMR | CEMP | CEMP Ratio to White | COVID MR | Non-Covid NMR | CEMP | CEMP Ratio to White |
| 18-39 | 0.003% | 0.018% | 16.81% |  | 0.005% | 0.033% | 14.55% | 0.87 | 0.004% | 0.019% | 22.22% | 1.32 | 0.003% | 0.008% | 40.00% | 2.38 |
| 40-49 | 0.008% | 0.073% | 10.33% |  | 0.020% | 0.141% | 14.44% | 1.40 | 0.020% | 0.068% | 28.57% | 2.77 | 0.021% | 0.050% | 42.11% | 4.08 |
| 50-59 | 0.025% | 0.179% | 13.95% |  | 0.044% | 0.351% | 12.56% | 0.90 | 0.023% | 0.129% | 18.18% | 1.30 | 0.023% | 0.180% | 12.96% | 0.93 |
| 60-69 | 0.057% | 0.516% | 11.10% |  | 0.123% | 0.838% | 14.66% | 1.32 | 0.076% | 0.352% | 21.43% | 1.93 | 0.043% | 0.273% | 15.87% | 1.43 |
| 70-79 | 0.130% | 1.303% | 9.95% |  | 0.206% | 1.871% | 10.99% | 1.10 | 0.319% | 0.935% | 34.12% | 3.43 | 0.125% | 0.720% | 17.39% | 1.75 |
| 80-89 | 0.237% | 3.164% | 7.49% |  | 0.438% | 3.412% | 12.85% | 1.72 | 0.374% | 2.697% | 13.86% | 1.85 | 0.071% | 2.449% | 2.88% | 0.39 |
| 90+ | 0.503% | 8.863% | 5.67% |  | 0.744% | 6.446% | 11.54% | 2.03 | 0.552% | 4.784% | 11.54% | 2.03 | 0.221% | 6.505% | 3.39% | 0.60 |
| 18-59 | 0.010% | 0.074% | 13.46% |  | 0.016% | 0.121% | 13.35% | 0.99 | 0.010% | 0.046% | 22.64% | 1.68 | 0.010% | 0.044% | 22.89% | 1.70 |
| 60+ | 0.132% | 1.616% | 8.15% |  | 0.211% | 1.678% | 12.54% | 1.54 | 0.189% | 0.912% | 20.78% | 2.55 | 0.076% | 0.775% | 9.75% | 1.20 |
| Total | 0.052% | 0.601% | 8.58% |  | 0.060% | 0.470% | 12.71% | 1.48 | 0.033% | 0.157% | 21.26% | 2.48 | 0.022% | 0.174% | 12.47% | 1.45 |

# Table App-4b. Mortality Rates by Age and Race/Ethnicity for Wisconsin, Indiana, and Illinois (Male)

COVID-19 MR, Non-COVID natural mortality rate, and CEMP for Wisconsin, Indiana, and Illinois males during indicated time periods. The Illinois data ends December 31, 2021. The last column in each panel for Black, Hispanic, and Other reports the ratio of CEMP to the corresponding CEMP for White. See Table App-4 for confidence intervals for all reported CEMP Ratio to White values. All panels. Data is for decedents so results are effectively weighted by mortality, not population. *, **, *** indicates p < .05, .01, and .001, respectively; significant results (at p < .05 or better) in boldface.

MR **=** $\frac{COVID deaths}{Population}$ ; Non-COVID Natural Mortality Rate (NMR) =$\frac{Non-Covid natural deaths}{Population}$ ; CEMP **=** $\frac{COVID deaths}{Non-COVID natural deaths}$

**Panel A. Pre-Vaccine Period (April-December 2020). Indiana, Wisconsin, and Illinois**

|  | **White** | | | | **Black** | | | | **Hispanic** | | | | **Other** | | | |
| --- | --- | --- | --- | --- | --- | --- | --- | --- | --- | --- | --- | --- | --- | --- | --- | --- |
| Age | COVID MR | Non-Covid NMR | CEMP |  | COVID MR | Non-Covid NMR | CEMP | CEMP Ratio to White | COVID MR | Non-Covid NMR | CEMP | CEMP Ratio to White | COVID MR | Non-Covid NMR | CEMP | CEMP Ratio to White |
| 18-39 | 0.002% | 0.037% | 4.84% |  | 0.013% | 0.086% | 15.06% | 3.12 | 0.017% | 0.031% | 56.80% | 11.75 | 0.004% | 0.019% | 24.07% | 4.98 |
| 40-49 | 0.012% | 0.151% | 7.92% |  | 0.065% | 0.399% | 16.17% | 2.04 | 0.100% | 0.137% | 72.76% | 9.19 | 0.020% | 0.085% | 24.10% | 3.04 |
| 50-59 | 0.037% | 0.471% | 7.79% |  | 0.164% | 0.907% | 18.08% | 2.32 | 0.278% | 0.335% | 82.97% | 10.66 | 0.058% | 0.249% | 23.28% | 2.99 |
| 60-69 | 0.146% | 1.172% | 12.46% |  | 0.512% | 2.380% | 21.51% | 1.73 | 0.701% | 0.792% | 88.49% | 7.10 | 0.224% | 0.587% | 38.27% | 3.07 |
| 70-79 | 0.483% | 2.593% | 18.62% |  | 1.054% | 3.894% | 27.06% | 1.45 | 1.316% | 1.671% | 78.72% | 4.23 | 0.512% | 1.280% | 40.05% | 2.15 |
| 80-89 | 1.326% | 6.302% | 21.05% |  | 2.067% | 7.222% | 28.62% | 1.36 | 2.314% | 4.372% | 52.92% | 2.51 | 1.129% | 3.424% | 32.98% | 1.57 |
| 90+ | 3.391% | 15.835% | 21.42% |  | 3.618% | 13.463% | 26.87% | 1.25 | 4.413% | 10.486% | 42.08% | 1.97 | 2.801% | 10.439% | 26.83% | 1.25 |
| 18-59 | 0.013% | 0.177% | 7.50% |  | 0.054% | 0.319% | 17.09% | 2.28 | 0.079% | 0.105% | 75.26% | 10.04 | 0.017% | 0.070% | 23.62% | 3.15 |
| 60+ | 0.500% | 2.711% | 18.43% |  | 0.902% | 3.571% | 25.25% | 1.37 | 1.087% | 1.543% | 70.44% | 3.82 | 0.457% | 1.276% | 35.78% | 1.94 |
| Total | 0.163% | 0.956% | 17.03% |  | 0.231% | 0.995% | 23.18% | 1.36 | 0.211% | 0.294% | 71.94% | 4.22 | 0.094% | 0.282% | 33.29% | 1.95 |

**Panel B. Early Vaccine Period (January-June 2021). Indiana, Wisconsin, and Illinois**

|  | **White** | | | | **Black** | | | | **Hispanic** | | | | **Other** | | | |
| --- | --- | --- | --- | --- | --- | --- | --- | --- | --- | --- | --- | --- | --- | --- | --- | --- |
| Age | COVID MR | Non-Covid NMR | CEMP |  | COVID MR | Non-Covid NMR | CEMP | CEMP Ratio to White | COVID MR | Non-Covid NMR | CEMP | CEMP Ratio to White | COVID MR | Non-Covid NMR | CEMP | CEMP Ratio to White |
| 18-39 | 0.001% | 0.026% | 5.75% |  | 0.004% | 0.056% | 7.17% | 1.25 | 0.006% | 0.022% | 28.10% | 4.89 | 0.002% | 0.011% | 18.18% | 3.16 |
| 40-49 | 0.008% | 0.104% | 7.96% |  | 0.025% | 0.234% | 10.85% | 1.36 | 0.038% | 0.081% | 47.65% | 5.99 | 0.006% | 0.045% | 13.64% | 1.71 |
| 50-59 | 0.025% | 0.295% | 8.44% |  | 0.081% | 0.587% | 13.73% | 1.63 | 0.091% | 0.224% | 40.72% | 4.82 | 0.033% | 0.165% | 20.00% | 2.37 |
| 60-69 | 0.071% | 0.771% | 9.16% |  | 0.174% | 1.430% | 12.18% | 1.33 | 0.250% | 0.525% | 47.63% | 5.20 | 0.071% | 0.378% | 18.66% | 2.04 |
| 70-79 | 0.198% | 1.725% | 11.47% |  | 0.296% | 2.532% | 11.68% | 1.02 | 0.441% | 1.174% | 37.59% | 3.28 | 0.210% | 0.735% | 28.57% | 2.49 |
| 80-89 | 0.423% | 4.033% | 10.49% |  | 0.551% | 4.316% | 12.76% | 1.22 | 0.658% | 2.809% | 23.42% | 2.23 | 0.392% | 2.231% | 17.55% | 1.67 |
| 90+ | 0.899% | 9.440% | 9.53% |  | 1.083% | 8.122% | 13.33% | 1.40 | 1.134% | 6.316% | 17.95% | 1.88 | 0.891% | 4.774% | 18.67% | 1.96 |
| 18-59 | 0.009% | 0.114% | 8.03% |  | 0.024% | 0.202% | 11.95% | 1.49 | 0.028% | 0.069% | 40.16% | 5.00 | 0.008% | 0.043% | 18.32% | 2.28 |
| 60+ | 0.181% | 1.747% | 10.34% |  | 0.269% | 2.200% | 12.22% | 1.18 | 0.356% | 1.021% | 34.90% | 3.37 | 0.163% | 0.766% | 21.32% | 2.06 |
| Total | 0.062% | 0.616% | 10.05% |  | 0.075% | 0.617% | 12.15% | 1.21 | 0.071% | 0.193% | 36.53% | 3.63 | 0.035% | 0.170% | 20.69% | 2.06 |

**Panel C. Delta Period (July-December 2021). Indiana, Wisconsin, and Illinois**

|  | **White** | | | | **Black** | | | | **Hispanic** | | | | **Other** | | | |
| --- | --- | --- | --- | --- | --- | --- | --- | --- | --- | --- | --- | --- | --- | --- | --- | --- |
| Age | COVID MR | Non-Covid NMR | CEMP |  | COVID MR | Non-Covid NMR | CEMP | CEMP Ratio to White | COVID MR | Non-Covid NMR | CEMP | CEMP Ratio to White | COVID MR | Non-Covid NMR | CEMP | CEMP Ratio to White |
| 18-39 | 0.007% | 0.026% | 25.68% |  | 0.015% | 0.057% | 26.77% | 1.04 | 0.013% | 0.023% | 56.25% | 2.19 | 0.006% | 0.018% | 32.69% | 1.27 |
| 40-49 | 0.034% | 0.107% | 31.64% |  | 0.053% | 0.241% | 22.16% | 0.70 | 0.055% | 0.092% | 60.31% | 1.91 | 0.016% | 0.056% | 29.09% | 0.92 |
| 50-59 | 0.072% | 0.311% | 23.27% |  | 0.107% | 0.609% | 17.52% | 0.75 | 0.087% | 0.208% | 41.61% | 1.79 | 0.021% | 0.174% | 12.12% | 0.52 |
| 60-69 | 0.142% | 0.805% | 17.70% |  | 0.184% | 1.588% | 11.57% | 0.65 | 0.149% | 0.568% | 26.25% | 1.48 | 0.045% | 0.378% | 11.96% | 0.68 |
| 70-79 | 0.264% | 1.841% | 14.32% |  | 0.241% | 2.422% | 9.96% | 0.70 | 0.238% | 1.110% | 21.45% | 1.50 | 0.143% | 0.824% | 17.37% | 1.21 |
| 80-89 | 0.497% | 4.279% | 11.61% |  | 0.425% | 4.577% | 9.28% | 0.80 | 0.487% | 2.739% | 17.80% | 1.53 | 0.246% | 2.250% | 10.93% | 0.94 |
| 90+ | 0.855% | 10.606% | 8.07% |  | 0.763% | 8.688% | 8.78% | 1.09 | 0.688% | 6.964% | 9.88% | 1.23 | 0.382% | 6.111% | 6.25% | 0.77 |
| 18-59 | 0.030% | 0.119% | 25.29% |  | 0.042% | 0.208% | 20.15% | 0.80 | 0.035% | 0.069% | 50.32% | 1.99 | 0.011% | 0.051% | 20.50% | 0.81 |
| 60+ | 0.248% | 1.867% | 13.27% |  | 0.238% | 2.299% | 10.37% | 0.78 | 0.215% | 1.035% | 20.76% | 1.56 | 0.104% | 0.817% | 12.70% | 0.96 |
| Total | 0.097% | 0.656% | 14.77% |  | 0.083% | 0.643% | 12.87% | 0.87 | 0.059% | 0.196% | 29.86% | 2.02 | 0.027% | 0.186% | 14.48% | 0.98 |

**Panel D. Omicron Period (January-June 2022). Indiana and Wisconsin**

|  | **White** | | | | **Black** | | | | **Hispanic** | | | | **Other** | | | |
| --- | --- | --- | --- | --- | --- | --- | --- | --- | --- | --- | --- | --- | --- | --- | --- | --- |
| Age | COVID MR | Non-Covid NMR | CEMP |  | COVID MR | Non-Covid NMR | CEMP | CEMP Ratio to White | COVID MR | Non-Covid NMR | CEMP | CEMP Ratio to White | COVID MR | Non-Covid NMR | CEMP | CEMP Ratio to White |
| 18-39 | 0.003% | 0.025% | 11.24% |  | 0.004% | 0.051% | 7.06% | 0.63 | 0.006% | 0.018% | 34.48% | 3.07 | 0.002% | 0.014% | 17.65% | 1.57 |
| 40-49 | 0.015% | 0.098% | 14.94% |  | 0.034% | 0.223% | 15.38% | 1.03 | 0.028% | 0.106% | 26.23% | 1.76 | 0.008% | 0.096% | 8.82% | 0.59 |
| 50-59 | 0.040% | 0.276% | 14.40% |  | 0.050% | 0.496% | 10.00% | 0.69 | 0.037% | 0.211% | 17.50% | 1.21 | 0.037% | 0.255% | 14.49% | 1.01 |
| 60-69 | 0.089% | 0.801% | 11.12% |  | 0.217% | 1.354% | 16.01% | 1.44 | 0.157% | 0.550% | 28.57% | 2.57 | 0.059% | 0.534% | 11.11% | 1.00 |
| 70-79 | 0.206% | 1.840% | 11.18% |  | 0.297% | 2.424% | 12.24% | 1.09 | 0.283% | 1.282% | 22.12% | 1.98 | 0.124% | 0.974% | 12.77% | 1.14 |
| 80-89 | 0.418% | 4.161% | 10.04% |  | 0.610% | 4.302% | 14.18% | 1.41 | 0.619% | 3.495% | 17.71% | 1.76 | 0.327% | 2.682% | 12.20% | 1.21 |
| 90+ | 0.911% | 9.997% | 9.11% |  | 1.177% | 7.401% | 15.91% | 1.75 | 1.235% | 7.937% | 15.56% | 1.71 | 0.906% | 7.553% | 12.00% | 1.32 |
| 18-59 | 0.015% | 0.107% | 14.14% |  | 0.019% | 0.172% | 10.95% | 0.77 | 0.016% | 0.067% | 23.53% | 1.66 | 0.009% | 0.064% | 13.33% | 0.94 |
| 60+ | 0.191% | 1.814% | 10.52% |  | 0.297% | 2.067% | 14.36% | 1.37 | 0.249% | 1.123% | 22.13% | 2.10 | 0.114% | 0.951% | 12.00% | 1.14 |
| Total | 0.069% | 0.628% | 10.95% |  | 0.070% | 0.523% | 13.45% | 1.23 | 0.042% | 0.185% | 22.58% | 2.06 | 0.024% | 0.191% | 12.38% | 1.13 |

# Table App-5. Confidence Intervals for the CEMP Ratios in text Table 2

Table provides same CEMP ratios (odds ratio for Black, Hispanic, or Other relative to White) as in text Table 2, but adds 95% confidence intervals, estimated using the logit model described in text, with race/ethnicity indicators without other covariates. The logit models are estimated by age group, separately for each time period.

|  | **April – December 2020** | | | | | | **January – June 2021** | | | | | |
| --- | --- | --- | --- | --- | --- | --- | --- | --- | --- | --- | --- | --- |
|  | **Black** | | **Hispanic** | | **Other** | | **Black** | | **Hispanic** | | **Other** | |
| Age | Odds ratio | 95% CI | Odds ratio | 95% CI | Odds ratio | 95% CI | Odds ratio | 95% CI | Odds ratio | 95% CI | Odds ratio | 95% CI |
| 18-39 | 3.02 | [2.19, 4.05] | 10.38 | [7.52, 13.95] | 4.30 | [2.54, 7.08] | 1.91 | [1.31, 2.88] | 3.88 | [2.59, 6.04] | 1.59 | [0.68, 3.90] |
| 40-49 | 2.26 | [1.81, 2.77] | 8.99 | [7.23, 10.95] | 3.58 | [2.33, 5.39] | 1.96 | [1.48, 2.60] | 5.79 | [4.36, 7.73] | 2.66 | [1.46, 4.83] |
| 50-59 | 2.35 | [2.07, 2.66] | 10.35 | [9.07, 11.82] | 3.16 | [2.41, 4.14] | 1.58 | [1.32, 1.87] | 4.89 | [4.03, 5.91] | 2.47 | [1.72, 3.51] |
| 60-69 | 1.74 | [1.60, 1.88] | 6.46 | [5.84, 7.11] | 2.67 | [2.24, 3.16] | 1.38 | [1.22, 1.57] | 4.81 | [4.15, 5.57] | 2.07 | [1.57, 2.71] |
| 70-79 | 1.52 | [1.41, 1.63] | 3.92 | [3.57, 4.33] | 2.06 | [1.78, 2.37] | 1.26 | [1.12, 1.42] | 3.19 | [2.75, 3.69] | 2.04 | [1.63, 2.55] |
| 80-89 | 1.26 | [1.17, 1.36] | 2.28 | [2.06, 2.53] | 1.56 | [1.35, 1.80] | 1.20 | [1.05, 1.37] | 2.06 | [1.72, 2.46] | 1.29 | [1.00, 1.67] |
| 90+ | 1.23 | [1.10, 1.37] | 1.76 | [1.50, 2.05] | 1.48 | [1.22, 1.79] | 1.18 | [0.97, 1.44] | 1.56 | [1.17, 2.08] | 1.40 | [0.97, 2.03] |
| 18-59 | 2.34 | [2.11, 2.58] | 9.57 | [8.60, 10.58] | 3.30 | [2.68, 4.04] | 1.68 | [1.46, 1.93] | 4.81 | [4.16, 5.58] | 2.32 | [1.74, 3.10] |
| 60+ | 1.35 | [1.30, 1.41] | 3.31 | [3.14, 3.49] | 1.82 | [1.68, 1.97] | 1.28 | [1.19, 1.37] | 3.05 | [2.80, 3.31] | 1.70 | [1.49, 1.95] |
| 18+ | 1.35 | [1.30, 1.40] | 3.70 | [3.54, 3.87] | 1.86 | [1.73, 2.00] | 1.32 | [1.25, 1.41] | 3.34 | [3.10, 3.58] | 1.77 | [1.57, 2.00] |

|  | **July-December 2021** | | | | | | **January – June 2022** | | | | | |
| --- | --- | --- | --- | --- | --- | --- | --- | --- | --- | --- | --- | --- |
|  | **Black** | | **Hispanic** | | **Other** | | **Black** | | **Hispanic** | | **Other** | |
| Age | Odds ratio | 95% CI | Odds ratio | 95% CI | Odds ratio | 95% CI | Odds ratio | 95% CI | Odds ratio | 95% CI | Odds ratio | 95% CI |
| 18-39 | 1.04 | [0.78, 1.26] | 1.79 | [1.27, 2.27] | 1.21 | [0.75, 1.92] | 0.74 | [0.41, 1.35] | 2.12 | [1.16, 3.87] | 1.92 | [0.81, 4.56] |
| 40-49 | 0.79 | [0.66, 0.98] | 1.76 | [1.41, 2.21] | 0.96 | [0.65, 1.64] | 1.15 | [0.77, 1.73] | 2.08 | [1.31, 3.33] | 1.60 | [0.81, 3.13] |
| 50-59 | 0.79 | [0.68, 0.90] | 1.59 | [1.31, 1.91] | 0.63 | [0.43, 0.95] | 0.78 | [0.58, 1.06] | 1.25 | [0.78, 1.98] | 0.97 | [0.58, 1.63] |
| 60-69 | 0.78 | [0.70, 0.87] | 1.59 | [1.38, 1.90] | 0.80 | [0.61, 1.11] | 1.39 | [1.15, 1.69] | 2.32 | [1.67, 3.22] | 1.17 | [0.74, 1.86] |
| 70-79 | 0.89 | [0.77, 0.98] | 1.35 | [1.14, 1.63] | 1.00 | [0.79, 1.32] | 1.09 | [0.88, 1.35] | 2.59 | [1.89, 3.54] | 1.42 | [0.95, 2.12] |
| 80-89 | 0.91 | [0.79, 1.05] | 1.26 | [1.02, 1.56] | 0.77 | [0.57, 1.07] | 1.55 | [1.22, 1.96] | 1.81 | [1.23, 2.66] | 0.80 | [0.46, 1.41] |
| 90+ | 1.11 | [0.88, 1.38] | 1.34 | [0.97, 1.85] | 0.97 | [0.65, 1.63] | 1.88 | [1.33, 2.66] | 1.96 | [1.09, 3.52] | 0.87 | [0.35, 2.15] |
| 18-59 | 0.85 | [0.76, 0.93] | 1.74 | [1.52, 1.96] | 0.87 | [0.70, 1.15] | 0.86 | [0.69, 1.08] | 1.67 | [1.26, 2.22] | 1.24 | [0.86, 1.79] |
| 60+ | 0.96 | [0.89, 1.02] | 1.51 | [1.39, 1.69] | 0.90 | [0.79, 1.08] | 1.44 | [1.29, 1.62] | 2.31 | [1.92, 2.77] | 1.17 | [0.90, 1.50] |
| 18+ | 1.03 | [0.97, 1.08] | 1.84 | [1.71, 1.99] | 0.96 | [0.86, 1.12] | 1.34 | [1.21, 1.48] | 2.25 | [1.93, 2.63] | 1.27 | [1.03, 1.57] |

# Table App-6. Multinomial Logit Model Separately for Men and Women

Table is similar to text Table 3, but shows results from the multinomial logit model separately for females and males.

**Panel A. Female**

|  | 18-59 | | | | 60+ | | | |
| --- | --- | --- | --- | --- | --- | --- | --- | --- |
| Category | Apr-Dec 2020 | Jan-Jun 2021 | Jul-Dec 2021 | Jan-Jun 2022 | Apr-Dec 2020 | Jan-Jun 2021 | Jul-Dec 2021 | Jan-Jun 2022 |
| Black | **2.48^***^** | **1.86^***^** | 0.86 | 1.00 | **1.30^***^** | **1.25^***^** | 0.97 | **1.32^**^** |
| Hispanic | **8.12^***^** | **4.18^***^** | 1.17 | **1.79^*^** | **2.67^***^** | **2.42^***^** | **1.35^***^** | **2.32^***^** |
| Other | **3.45^***^** | **2.33^***^** | 0.92 | **1.76^*^** | **1.70^***^** | **1.36^**^** | 0.79 | 1.19 |
| Age bin |  |  |  |  |  |  |  |  |
| 2 | 1.08 | 1.15 | 0.90 | 0.74 | **1.30^***^** | 1.09 | **0.76^***^** | 0.91 |
| 3 | **1.41^**^** | 1.19 | **0.75^**^** | 0.79 | **1.45^***^** | 0.97 | **0.52^***^** | **0.69^***^** |
| 4 |  |  |  |  | **1.46^***^** | **0.87^**^** | **0.38^***^** | **0.53^***^** |
| zip SES Quartile |  |  |  |  |  |  |  |  |
| 2 | 1.30 | 1.01 | 1.05 | 0.86 | **1.15^***^** | **1.13^*^** | **1.25^***^** | 1.06 |
| 3 | 1.18 | 1.19 | 1.19 | 0.87 | **1.15^***^** | **1.19^***^** | **1.24^***^** | **1.16^*^** |
| 4 | **1.30^*^** | 1.23 | 1.13 | 0.79 | **1.24^***^** | **1.29^***^** | **1.29^***^** | **1.16^*^** |
| Education Level |  |  |  |  |  |  |  |  |
| Unknown | 0.91 | 0.79 | 1.17 | 1.03 | **1.58^***^** | 1.18 | **1.38^*^** | 1.27 |
| No High School | 0.91 | 0.79 | 1.18 | 1.06 | **1.13^***^** | **1.18^**^** | **1.65^***^** | **1.66^***^** |
| High School | 0.83 | 1.15 | **1.54^***^** | 1.07 | 1.04 | **1.17^**^** | **1.37^***^** | **1.56^***^** |
| Associate/Some College | **0.81^*^** | **1.36^*^** | **1.58^***^** | 1.33 | 1.02 | 1.07 | **1.32^***^** | **1.45^***^** |

**Panel B. Male**

|  | 18-59 | | | | 60+ | | | |
| --- | --- | --- | --- | --- | --- | --- | --- | --- |
| Category | Apr-Dec 2020 | Jan-Jun 2021 | Jul-Dec 2021 | Jan-Jun 2022 | Apr-Dec 2020 | Jan-Jun 2021 | Jul-Dec 2021 | Jan-Jun 2022 |
| Black | **2.08^***^** | **1.76^***^** | **0.83^*^** | 0.91 | **1.36^***^** | **1.18^**^** | **0.71^***^** | 1.18 |
| Hispanic | **9.20^***^** | **5.94^***^** | **2.18^***^** | **1.81^**^** | **3.80^***^** | **3.45^***^** | **1.52^***^** | **1.99^***^** |
| Other | **3.09^***^** | **2.36^***^** | 0.82 | 0.92 | **1.94^***^** | **2.14^***^** | 0.99 | 1.11 |
| Age bin |  |  |  |  |  |  |  |  |
| 2 | **1.27^*^** | **1.45^**^** | 1.07 | 1.24 | **1.34^***^** | **1.17^***^** | **0.85^***^** | 1.01 |
| 3 | **1.35^***^** | **1.51^***^** | **0.79^***^** | 1.13 | **1.46^***^** | 1.06 | **0.70^***^** | 0.93 |
| 4 |  |  |  |  | **1.46^***^** | 0.99 | **0.49^***^** | **0.86^*^** |
| zip SES Quartile |  |  |  |  |  |  |  |  |
| 2 | 0.92 | 1.19 | 0.91 | 0.91 | **1.11^***^** | 1.09 | **1.14^***^** | 1.07 |
| 3 | 1.15 | 1.20 | 0.91 | 1.05 | **1.16^***^** | **1.14^**^** | **1.15^***^** | 1.08 |
| 4 | **1.32^***^** | 0.87 | **0.82^*^** | 0.71 | **1.19^***^** | 1.07 | 1.06 | **1.18^*^** |
| Education Level |  |  |  |  |  |  |  |  |
| Unknown | 1.16 | 1.12 | 0.78 | 0.80 | **1.34^***^** | 0.84 | 1.05 | 1.01 |
| No High School | **0.71^***^** | **0.67^**^** | 0.88 | 0.73 | 1.04 | 1.05 | **1.41^***^** | **1.28^***^** |
| High School | **0.85^*^** | 0.85 | **1.30^***^** | 0.88 | 1.00 | **1.09^*^** | **1.27^***^** | **1.20^**^** |
| Associate/Some College | **0.84^*^** | 1.07 | **1.42^***^** | 1.09 | 0.96 | 1.10 | **1.16^***^** | 1.12 |

# Table App-7. State-Specific Results from Racial/Ethnic Disparities in COVID Mortality Rates: Multivariate Logit Analysis

Table is similar to Table 3 in text, but reports results separately for Indiana, Wisconsin, and Illinois. Data for Illinois covers only the first three periods. *, **, *** indicates p < .05, .01, and .001, respectively; significant results (at p < .05 or better) in boldface.

**Panel A. Indiana**

|  | 18-59 | | | | 60+ | | | |
| --- | --- | --- | --- | --- | --- | --- | --- | --- |
| Category | Apr-Dec 2020 | Jan-Jun 2021 | Jul-Dec 2021 | Jan-Jun 2022 | Apr-Dec 2020 | Jan-Jun 2021 | Jul-Dec 2021 | Jan-Jun 2022 |
| Black | **2.45^***^** | **1.51^*^** | 1.00 | 0.92 | **1.24^***^** | **1.22^**^** | 0.07 | 1.20 |
| Hispanic | **6.98^***^** | **6.72^***^** | **2.64^***^** | **1.81^***^** | **2.12^***^** | **2.63^***^** | **1.68^***^** | **1.85^***^** |
| Other | **2.56^**^** | **3.01^**^** | 0.65 | 1.02 | **2.09^***^** | **1.54^*^** | **0.64** | 1.02 |
| Male | 1.04 | 0.91 | 1.09 | 1.17 | **1.15^***^** | **1.18^***^** | **1.13^***^** | **1.19^***^** |
| Age bin |  |  |  |  |  |  |  |  |
| 2 | 1.35 | **1.68^*^** | 1.12 | 1.13 | **1.38^***^** | **1.23^***^** | **0.79^***^** | 0.98 |
| 3 | **1.63^***^** | **1.96^**^** | 0.89 | 1.09 | **1.65^***^** | **1.22^***^** | **0.62^***^** | **0.87^*^** |
| 4 |  |  |  |  | **1.70^***^** | 1.09 | **0.44^***^** | **0.67^***^** |
| zip SES Quartile |  |  |  |  |  |  |  |  |
| 2 | 0.85 | 1.47 | 0.84 | 0.87 | **1.11^**^** | 1.14 | 1.13 | 1.11 |
| 3 | 0.84 | 1.29 | 0.96 | 0.99 | **1.12^**^** | 1.14 | 1.07 | 1.10 |
| 4 | 0.90 | 0.95 | 0.85 | 0.75 | **1.13^***^** | 1.03 | 1.01 | 1.06 |
| Education Level |  |  |  |  |  |  |  |  |
| Unknown | 1.21 | 1.30 | 0.56 | 0.80 | **1.68^***^** | 1.13 | 1.14 | 0.93 |
| No High School | **0.69^*^** | 0.72 | **0.73^*^** | 0.78 | **1.10^*^** | 1.17 | 1.37 | 1.25 |
| High School | **0.70^*^** | 0.76 | 1.01 | 0.89 | 1.04 | 1.15 | 1.20 | 1.21 |
| Associate/Some College | 0.82 | 0.98 | 0.98 | 1.22 | 1.07 | 1.18 | 1.23 | 1.14 |

**Panel B. Wisconsin**

|  | 18-59 | | | | 60+ | | | |
| --- | --- | --- | --- | --- | --- | --- | --- | --- |
| Category | Apr-Dec 2020 | Jan-Jun 2021 | Jul-Dec 2021 | Jan-Jun 2022 | Apr-Dec 2020 | Jan-Jun 2021 | Jul-Dec 2021 | Jan-Jun 2022 |
| Black | **1.64^**^** | 1.39 | 0.88 | 1.03 | **1.40^***^** | **1.43^**^** | 1.13 | **1.34^*^** |
| Hispanic | **9.12^***^** | **3.92^***^** | **2.11^***^** | **1.95^**^** | **3.35^***^** | **2.49^***^** | **1.87^***^** | **2.65^***^** |
| Other | **3.09^***^** | **3.33^***^** | 0.91 | 1.54 | **1.78^***^** | **1.74^**^** | **1.47^**^** | 1.36 |
| Male | 1.12 | 1.32 | 1.21**^*^** | 0.82 | **1.24^***^** | **1.23^***^** | **1.19^***^** | **1.29^***^** |
| Age bin |  |  |  |  |  |  |  |  |
| 2 | 0.87 | 1.45 | 1.19 | 0.77 | **1.48^***^** | 1.15 | **0.79^***^** | 0.97 |
| 3 | 1.01 | 1.24 | 0.83 | 0.80 | **1.60^***^** | **1.23^*^** | **0.64^***^** | **0.77^***^** |
| 4 |  |  |  |  | **1.71^***^** | 1.16 | **0.48^***^** | **0.71^***^** |
| zip SES Quartile |  |  |  |  |  |  |  |  |
| 2 | 1.15 | 1.31 | 0.79 | 0.89 | 1.06 | 0.96 | 1.09 | 0.98 |
| 3 | 1.09 | 0.78 | **0.74^*^** | 0.87 | 1.01 | **0.82^*^** | 0.96 | 0.97 |
| 4 | **1.44^*^** | 1.15 | 0.82 | 0.64 | **1.16^**^** | 0.88 | 0.94 | 1.17 |
| Education Level |  |  |  |  |  |  |  |  |
| Unknown | 0.87 | 0.76 | 2.09**^*^** | 1.08 | 1.13 | 1.27 | 1.19 | 1.33 |
| No High School | 0.88 | 0.69 | 0.74 | 1.00 | 1.18**^**^** | **1.27^*^** | **1.35^***^** | **1.56^***^** |
| High School | 0.94 | 0.87 | **1.41^*^** | 1.09 | 1.09 | **1.25^**^** | **1.34^***^** | **1.40^***^** |
| Associate/Some College | 0.81 | 0.89 | **1.58^**^** | 1.16 | 0.97 | 1.09 | **1.24^**^** | **1.35^***^** |

**Panel C. Illinois**

|  | 18-59 | | | 60+ | | |
| --- | --- | --- | --- | --- | --- | --- |
| Category | Apr-Dec 2020 | Jan-Jun 2021 | Jul-Dec 2021 | Apr-Dec 2020 | Jan-Jun 2021 | Jul-Dec 2021 |
| Black | **2.14^***^** | **1.78^***^** | **0.85^*^** | **1.35^***^** | 1.16 | **0.82^***^** |
| Hispanic | **8.68^***^** | **4.69^***^** | **1.77^***^** | **3.51^***^** | **3.00^***^** | **1.39^***^** |
| Other | **3.40^***^** | **1.95^***^** | 0.88 | **1.87^***^** | **1.86^***^** | 0.83 |
| Male | **1.19^**^** | **1.17^*^** | **1.23^***^** | **1.23^***^** | **1.35^***^** | **1.15^***^** |
| Age bin |  |  |  |  |  |  |
| 2 | **1.25^*^** | 1.24 | 0.86 | **1.25^***^** | **1.10^*^** | **0.85^***^** |
| 3 | **1.38^***^** | **1.26^*^** | **0.67^***^** | **1.32^***^** | **0.89^**^** | **0.61^***^** |
| 4 |  |  |  | **1.30^***^** | **0.81^***^** | **0.42^***^** |
| zip SES Quartile |  |  |  |  |  |  |
| 2 | 1.13 | 0.96 | 1.19 | **1.16^***^** | **1.15^**^** | **1.23^***^** |
| 3 | **1.39^**^** | 1.21 | 1.24**^*^** | **1.17^***^** | **1.24^***^** | **1.38^***^** |
| 4 | **1.52^***^** | 0.95 | 1.19 | **1.19^***^** | **1.22^***^** | **1.38^***^** |
| Education Level |  |  |  |  |  |  |
| Unknown | 1.13 | 1.02 | **0.48^*^** | **1.49^***^** | 0.86 | 1.07 |
| No High School | 0.84 | **0.71^*^** | 1.01 | 1.07 | 1.00 | **1.32^***^** |
| High School | 0.91 | 1.08 | **1.44^***^** | 1.00 | **1.08^*^** | **1.20^***^** |
| Associate/Some College | 0.86 | 1.34 | **1.64^***^** | 0.98 | 1.07 | **1.13^*^** |

# Table App-8. Alternate CEMP Rates and CEMP Ratios Using 2019 Natural Mortality in Denominator

Table is similar to text Table 2 but replaces Non-COVID natural mortality rate for indicated period with total natural mortality rate in 2019, and measures CEMP using 2019 natural mortality rate as the denominator (“CEMP-2019”). Last column in each panel for Black, Hispanic, and Other reports the ratio of CEMP-2019 to the corresponding CEMP-2019 for White. *, **, *** indicates p < .05, .01, and .001, respectively; significant results (at p < .05 or better) in **boldface**.

COVID MR **=** $\frac{COVID deaths}{Population}$ ; 2019 Natural Mortality Rate (NMR) =$\frac{natural deaths in 2019}{Population}$ ; CEMP-2019 **=** $\frac{COVID deaths}{2019 natural deaths}$

**Panel A. Pre-Vaccine Period (April-December 2020). Indiana, Wisconsin, and Illinois**

|  | **White** | | | | **Black** | | | | **Hispanic** | | | | **Other** | | | |
| --- | --- | --- | --- | --- | --- | --- | --- | --- | --- | --- | --- | --- | --- | --- | --- | --- |
| Age | COVID MR | 2019 NMR | CEMP-2019 |  | COVID MR | 2019 NMR | CEMP-2019 | CEMP Ratio to White | COVID MR | 2019 NMR | CEMP-2019 | CEMP Ratio to White | COVID MR | 2019 NMR | CEMP-2019 | CEMP Ratio to White |
| 18-39 | 0.002% | 0.031% | 5.11% |  | 0.011% | 0.067% | 16.97% | 3.32 | 0.012% | 0.024% | 52.59% | 10.30 | 0.004% | 0.013% | 28.21% | 5.52 |
| 40-49 | 0.009% | 0.124% | 7.35% |  | 0.051% | 0.280% | 18.16% | 2.47 | 0.067% | 0.100% | 67.08% | 9.12 | 0.015% | 0.059% | 25.83% | 3.51 |
| 50-59 | 0.029% | 0.373% | 7.90% |  | 0.132% | 0.677% | 19.48% | 2.47 | 0.198% | 0.246% | 80.71% | 10.22 | 0.044% | 0.153% | 28.74% | 3.64 |
| 60-69 | 0.112% | 0.902% | 12.42% |  | 0.378% | 1.614% | 23.42% | 1.89 | 0.500% | 0.587% | 85.12% | 6.85 | 0.149% | 0.407% | 36.55% | 2.94 |
| 70-79 | 0.373% | 2.104% | 17.73% |  | 0.795% | 2.705% | 29.38% | 1.66 | 0.960% | 1.318% | 72.90% | 4.11 | 0.371% | 0.988% | 37.55% | 2.12 |
| 80-89 | 1.051% | 5.403% | 19.46% |  | 1.539% | 5.786% | 26.60% | 1.37 | 1.753% | 3.720% | 47.12% | 2.42 | 0.936% | 2.640% | 35.45% | 1.82 |
| 90+ | 2.841% | 14.475% | 19.63% |  | 2.914% | 11.200% | 26.02% | 1.33 | 2.892% | 7.256% | 39.86% | 2.03 | 2.700% | 7.389% | 36.54% | 1.86 |
| 18-59 | 0.011% | 0.143% | 7.48% |  | 0.045% | 0.242% | 18.75% | 2.51 | 0.055% | 0.078% | 71.47% | 9.55 | 0.013% | 0.047% | 27.87% | 3.72 |
| 60+ | 0.449% | 2.520% | 17.81% |  | 0.728% | 2.766% | 26.33% | 1.48 | 0.826% | 1.304% | 63.34% | 3.56 | 0.367% | 1.005% | 36.52% | 2.05 |
| Total | 0.154% | 0.922% | 16.73% |  | 0.204% | 0.830% | 24.63% | 1.47 | 0.164% | 0.250% | 65.51% | 3.92 | 0.081% | 0.231% | 35.10% | 2.10 |

**Panel B. Early Vaccine Period (January-June 2021). Indiana, Wisconsin, and Illinois**

|  | **White** | | | | **Black** | | | | **Hispanic** | | | | **Other** | | | |
| --- | --- | --- | --- | --- | --- | --- | --- | --- | --- | --- | --- | --- | --- | --- | --- | --- |
| Age | COVID MR | 2019 NMR | CEMP-2019 |  | COVID MR | 2019 NMR | CEMP-2019 | CEMP Ratio to White | COVID MR | 2019 NMR | CEMP-2019 | CEMP Ratio to White | COVID MR | 2019 NMR | CEMP-2019 | CEMP Ratio to White |
| 18-39 | 0.001% | 0.021% | 6.06% |  | 0.006% | 0.047% | 11.66% | 1.92 | 0.004% | 0.019% | 21.39% | 3.53 | 0.001% | 0.009% | 11.76% | 1.94 |
| 40-49 | 0.006% | 0.087% | 7.01% |  | 0.026% | 0.181% | 14.54% | 2.07 | 0.026% | 0.067% | 38.89% | 5.54 | 0.007% | 0.043% | 16.09% | 2.29 |
| 50-59 | 0.020% | 0.262% | 7.54% |  | 0.060% | 0.468% | 12.88% | 1.71 | 0.067% | 0.154% | 43.31% | 5.74 | 0.024% | 0.121% | 19.90% | 2.64 |
| 60-69 | 0.054% | 0.611% | 8.83% |  | 0.135% | 1.068% | 12.69% | 1.44 | 0.177% | 0.368% | 48.06% | 5.44 | 0.052% | 0.256% | 20.45% | 2.32 |
| 70-79 | 0.149% | 1.437% | 10.40% |  | 0.253% | 1.796% | 14.11% | 1.36 | 0.327% | 0.839% | 38.98% | 3.75 | 0.135% | 0.588% | 22.93% | 2.20 |
| 80-89 | 0.317% | 3.732% | 8.49% |  | 0.427% | 3.951% | 10.79% | 1.27 | 0.491% | 2.501% | 19.64% | 2.31 | 0.243% | 1.678% | 14.51% | 1.71 |
| 90+ | 0.709% | 9.926% | 7.14% |  | 0.732% | 7.891% | 9.27% | 1.30 | 0.710% | 4.890% | 14.52% | 2.03 | 0.629% | 4.832% | 13.03% | 1.82 |
| 18-59 | 0.007% | 0.100% | 7.28% |  | 0.022% | 0.165% | 13.08% | 1.80 | 0.019% | 0.052% | 37.17% | 5.11 | 0.006% | 0.035% | 17.66% | 2.43 |
| 60+ | 0.151% | 1.727% | 8.73% |  | 0.226% | 1.862% | 12.14% | 1.39 | 0.267% | 0.848% | 31.43% | 3.60 | 0.113% | 0.627% | 18.09% | 2.07 |
| Total | 0.054% | 0.633% | 8.57% |  | 0.069% | 0.560% | 12.35% | 1.44 | 0.054% | 0.164% | 32.99% | 3.85 | 0.027% | 0.149% | 18.01% | 2.10 |

**Panel C. Delta Period (July-December 2021). Indiana, Wisconsin, and Illinois**

|  | **White** | | | | **Black** | | | | **Hispanic** | | | | **Other** | | | |
| --- | --- | --- | --- | --- | --- | --- | --- | --- | --- | --- | --- | --- | --- | --- | --- | --- |
| Age | COVID MR | 2019 NMR | CEMP-2019 |  | COVID MR | 2019 NMR | CEMP-2019 | CEMP Ratio to White | COVID MR | 2019 NMR | CEMP-2019 | CEMP Ratio to White | COVID MR | 2019 NMR | CEMP-2019 | CEMP Ratio to White |
| 18-39 | 0.006% | 0.021% | 27.09% |  | 0.014% | 0.044% | 32.01% | 1.18 | 0.008% | 0.015% | 57.79% | 2.13 | 0.004% | 0.008% | 53.33% | 1.97 |
| 40-49 | 0.026% | 0.081% | 32.47% |  | 0.047% | 0.195% | 24.26% | 0.75 | 0.036% | 0.066% | 53.93% | 1.66 | 0.012% | 0.037% | 31.58% | 0.97 |
| 50-59 | 0.057% | 0.247% | 23.12% |  | 0.086% | 0.448% | 19.21% | 0.83 | 0.060% | 0.174% | 34.40% | 1.49 | 0.017% | 0.090% | 19.18% | 0.83 |
| 60-69 | 0.115% | 0.605% | 19.00% |  | 0.165% | 1.082% | 15.24% | 0.80 | 0.121% | 0.400% | 30.31% | 1.60 | 0.042% | 0.279% | 14.91% | 0.78 |
| 70-79 | 0.212% | 1.409% | 15.02% |  | 0.237% | 1.841% | 12.89% | 0.86 | 0.186% | 0.919% | 20.25% | 1.35 | 0.092% | 0.675% | 13.58% | 0.90 |
| 80-89 | 0.367% | 3.592% | 10.21% |  | 0.365% | 3.883% | 9.41% | 0.92 | 0.328% | 2.501% | 13.13% | 1.29 | 0.160% | 1.784% | 8.96% | 0.88 |
| 90+ | 0.651% | 9.787% | 6.65% |  | 0.593% | 7.673% | 7.73% | 1.16 | 0.552% | 5.061% | 10.91% | 1.64 | 0.386% | 5.238% | 7.36% | 1.11 |
| 18-59 | 0.024% | 0.095% | 25.44% |  | 0.037% | 0.162% | 22.54% | 0.89 | 0.023% | 0.053% | 43.97% | 1.73 | 0.008% | 0.028% | 28.46% | 1.12 |
| 60+ | 0.206% | 1.688% | 12.18% |  | 0.225% | 1.868% | 12.06% | 0.99 | 0.173% | 0.894% | 19.35% | 1.59 | 0.079% | 0.689% | 11.45% | 0.94 |
| Total | 0.084% | 0.617% | 13.55% |  | 0.081% | 0.560% | 14.39% | 1.06 | 0.044% | 0.171% | 25.87% | 1.91 | 0.022% | 0.155% | 13.94% | 1.03 |

**Panel D. Omicron Period (January-June 2022). Indiana and Wisconsin**

|  | **White** | | | | **Black** | | | | **Hispanic** | | | | **Other** | | | |
| --- | --- | --- | --- | --- | --- | --- | --- | --- | --- | --- | --- | --- | --- | --- | --- | --- |
| Age | COVID MR | 2019 NMR | CEMP-2019 |  | COVID MR | 2019 NMR | CEMP-2019 | CEMP Ratio to White | COVID MR | 2019 NMR | CEMP-2019 | CEMP Ratio to White | COVID MR | 2019 NMR | CEMP-2019 | CEMP Ratio to White |
| 18-39 | 0.003% | 0.023% | 12.70% |  | 0.004% | 0.046% | 9.21% | 0.73 | 0.005% | 0.018% | 30.19% | 2.38 | 0.003% | 0.009% | 33.33% | 2.62 |
| 40-49 | 0.011% | 0.094% | 11.79% |  | 0.027% | 0.186% | 14.54% | 1.23 | 0.024% | 0.075% | 31.71% | 2.69 | 0.015% | 0.054% | 27.50% | 2.33 |
| 50-59 | 0.032% | 0.273% | 11.83% |  | 0.047% | 0.498% | 9.37% | 0.79 | 0.031% | 0.151% | 20.18% | 1.71 | 0.030% | 0.153% | 19.54% | 1.65 |
| 60-69 | 0.073% | 0.631% | 11.54% |  | 0.165% | 1.105% | 14.96% | 1.30 | 0.117% | 0.423% | 27.65% | 2.40 | 0.050% | 0.288% | 17.50% | 1.52 |
| 70-79 | 0.165% | 1.485% | 11.11% |  | 0.244% | 1.946% | 12.55% | 1.13 | 0.302% | 1.029% | 29.38% | 2.64 | 0.125% | 0.562% | 22.22% | 2.00 |
| 80-89 | 0.310% | 3.810% | 8.14% |  | 0.502% | 4.282% | 11.73% | 1.44 | 0.478% | 2.896% | 16.49% | 2.03 | 0.178% | 1.780% | 10.00% | 1.23 |
| 90+ | 0.630% | 10.043% | 6.28% |  | 0.861% | 7.677% | 11.21% | 1.79 | 0.786% | 5.925% | 13.27% | 2.11 | 0.404% | 4.523% | 8.93% | 1.42 |
| 18-59 | 0.013% | 0.105% | 11.92% |  | 0.017% | 0.165% | 10.60% | 0.89 | 0.013% | 0.051% | 26.23% | 2.20 | 0.009% | 0.039% | 23.65% | 1.98 |
| 60+ | 0.159% | 1.743% | 9.12% |  | 0.248% | 1.909% | 12.97% | 1.42 | 0.218% | 0.966% | 22.59% | 2.48 | 0.092% | 0.595% | 15.51% | 1.70 |
| Total | 0.060% | 0.636% | 9.43% |  | 0.065% | 0.523% | 12.37% | 1.31 | 0.038% | 0.160% | 23.60% | 2.50 | 0.023% | 0.129% | 17.59% | 1.86 |

# Table App-9. Comparison of CEMP and CEMP-2019 Rates and Ratios

Table compares CEMP and CEMP ratios (from text Table 2) and CEMP-2019 rates and ratios (from previous table).

**Panel A. Pre-Vaccine Period (April-December 2020). Indiana, Wisconsin, and Illinois**

|  | **White** | | **Black** | | | | **Hispanic** | | | | **Other** | | | |
| --- | --- | --- | --- | --- | --- | --- | --- | --- | --- | --- | --- | --- | --- | --- |
| Age | CEMP | CEMP-2019 | CEMP | CEMP-2019 | CEMP Ratio to White | CEMP-2019 Ratio to White | CEMP | CEMP-2019 | CEMP Ratio to White | CEMP-2019 Ratio to White | CEMP | CEMP-2019 | CEMP Ratio to White | CEMP-2019 Ratio to White |
| 18-39 | 4.873% | 5.106% | 14.714% | 16.969% | 3.02 | 3.32 | 50.575% | 52.590% | 10.38 | 10.30 | 20.952% | 28.205% | 4.30 | 5.52 |
| 40-49 | 6.979% | 7.354% | 15.799% | 18.163% | 2.26 | 2.47 | 62.731% | 67.079% | 8.99 | 9.12 | 25.000% | 25.833% | 3.58 | 3.51 |
| 50-59 | 7.611% | 7.901% | 17.880% | 19.480% | 2.35 | 2.47 | 78.809% | 80.709% | 10.35 | 10.22 | 24.068% | 28.745% | 3.16 | 3.64 |
| 60-69 | 11.790% | 12.424% | 20.463% | 23.422% | 1.74 | 1.89 | 76.178% | 85.121% | 6.46 | 6.85 | 31.488% | 36.546% | 2.67 | 2.94 |
| 70-79 | 17.064% | 17.733% | 25.856% | 29.376% | 1.52 | 1.66 | 66.959% | 72.901% | 3.92 | 4.11 | 35.085% | 37.553% | 2.06 | 2.12 |
| 80-89 | 19.536% | 19.459% | 24.643% | 26.600% | 1.26 | 1.37 | 44.619% | 47.115% | 2.28 | 2.42 | 30.521% | 35.447% | 1.56 | 1.82 |
| 90+ | 19.196% | 19.630% | 23.611% | 26.015% | 1.23 | 1.33 | 33.691% | 39.855% | 1.76 | 2.03 | 28.358% | 36.538% | 1.48 | 1.86 |
| 18-59 | 7.178% | 7.482% | 16.822% | 18.752% | 2.34 | 2.51 | 68.693% | 71.471% | 9.57 | 9.55 | 23.664% | 27.865% | 3.30 | 3.72 |
| 60+ | 17.392% | 17.805% | 23.518% | 26.331% | 1.35 | 1.48 | 57.574% | 63.344% | 3.31 | 3.56 | 31.676% | 36.524% | 1.82 | 2.05 |
| Total | 16.310% | 16.729% | 22.025% | 24.635% | 1.35 | 1.47 | 60.424% | 65.515% | 3.70 | 3.92 | 30.338% | 35.103% | 1.86 | 2.10 |

**Panel B. Early Vaccine Period (January-June 2021). Indiana, Wisconsin, and Illinois**

|  | **White** | | **Black** | | | | **Hispanic** | | | | **Other** | | | |
| --- | --- | --- | --- | --- | --- | --- | --- | --- | --- | --- | --- | --- | --- | --- |
| Age | CEMP | CEMP-2019 | CEMP | CEMP-2019 | CEMP Ratio to White | CEMP-2019 Ratio to White | CEMP | CEMP-2019 | CEMP Ratio to White | CEMP-2019 Ratio to White | CEMP | CEMP-2019 | CEMP Ratio to White | CEMP-2019 Ratio to White |
| 18-39 | 5.855% | 6.061% | 11.494% | 11.655% | 1.96 | 1.92 | 23.757% | 21.393% | 4.06 | 3.53 | 9.524% | 11.765% | 1.63 | 1.94 |
| 40-49 | 7.011% | 7.015% | 13.623% | 14.537% | 1.94 | 2.07 | 39.924% | 38.889% | 5.69 | 5.54 | 18.421% | 16.092% | 2.63 | 2.29 |
| 50-59 | 8.136% | 7.541% | 12.649% | 12.883% | 1.55 | 1.71 | 39.381% | 43.311% | 4.84 | 5.74 | 19.500% | 19.898% | 2.40 | 2.64 |
| 60-69 | 8.659% | 8.831% | 11.810% | 12.688% | 1.36 | 1.44 | 41.047% | 48.065% | 4.74 | 5.44 | 17.729% | 20.447% | 2.05 | 2.32 |
| 70-79 | 10.240% | 10.402% | 12.726% | 14.109% | 1.24 | 1.36 | 32.622% | 38.981% | 3.19 | 3.75 | 20.905% | 22.931% | 2.04 | 2.20 |
| 80-89 | 9.320% | 8.491% | 11.366% | 10.795% | 1.22 | 1.27 | 19.384% | 19.636% | 2.08 | 2.31 | 11.765% | 14.512% | 1.26 | 1.71 |
| 90+ | 8.057% | 7.138% | 9.471% | 9.273% | 1.18 | 1.30 | 12.827% | 14.516% | 1.59 | 2.03 | 11.071% | 13.025% | 1.37 | 1.82 |
| 18-59 | 7.631% | 7.276% | 12.704% | 13.077% | 1.66 | 1.80 | 36.491% | 37.171% | 4.78 | 5.11 | 17.404% | 17.665% | 2.28 | 2.43 |
| 60+ | 9.175% | 8.729% | 11.690% | 12.139% | 1.27 | 1.39 | 28.021% | 31.425% | 3.05 | 3.60 | 15.525% | 18.092% | 1.69 | 2.07 |
| Total | 9.011% | 8.575% | 11.918% | 12.351% | 1.32 | 1.44 | 30.175% | 32.994% | 3.35 | 3.85 | 15.845% | 18.010% | 1.76 | 2.10 |

**Panel C. Delta Period (July-December 2021). Indiana, Wisconsin, and Illinois**

|  | **White** | | **Black** | | | | **Hispanic** | | | | **Other** | | | |
| --- | --- | --- | --- | --- | --- | --- | --- | --- | --- | --- | --- | --- | --- | --- |
| Age | CEMP | CEMP-2019 | CEMP | CEMP-2019 | CEMP Ratio to White | CEMP-2019 Ratio to White | CEMP | CEMP-2019 | CEMP Ratio to White | CEMP-2019 Ratio to White | CEMP | CEMP-2019 | CEMP Ratio to White | CEMP-2019 Ratio to White |
| 18-39 | 26.002% | 27.087% | 26.008% | 32.010% | 1.00 | 1.18 | 47.090% | 57.792% | 1.81 | 2.13 | 31.169% | 53.333% | 1.20 | 1.97 |
| 40-49 | 28.352% | 32.471% | 22.527% | 24.260% | 0.79 | 0.75 | 49.827% | 53.933% | 1.76 | 1.66 | 27.907% | 31.579% | 0.98 | 0.97 |
| 50-59 | 22.451% | 23.118% | 17.615% | 19.213% | 0.78 | 0.83 | 35.759% | 34.400% | 1.59 | 1.49 | 13.930% | 19.178% | 0.62 | 0.83 |
| 60-69 | 17.445% | 18.997% | 13.602% | 15.238% | 0.78 | 0.80 | 27.493% | 30.312% | 1.58 | 1.60 | 14.697% | 14.912% | 0.84 | 0.78 |
| 70-79 | 13.526% | 15.021% | 12.073% | 12.895% | 0.89 | 0.86 | 18.431% | 20.246% | 1.36 | 1.35 | 13.721% | 13.580% | 1.01 | 0.90 |
| 80-89 | 10.111% | 10.209% | 9.278% | 9.408% | 0.92 | 0.92 | 12.785% | 13.134% | 1.26 | 1.29 | 7.807% | 8.955% | 0.77 | 0.88 |
| 90+ | 6.832% | 6.650% | 7.614% | 7.732% | 1.11 | 1.16 | 9.251% | 10.909% | 1.35 | 1.64 | 6.738% | 7.364% | 0.99 | 1.11 |
| 18-59 | 24.133% | 25.441% | 20.322% | 22.537% | 0.84 | 0.89 | 42.231% | 43.974% | 1.75 | 1.73 | 20.879% | 28.464% | 0.87 | 1.12 |
| 60+ | 11.693% | 12.177% | 11.335% | 12.056% | 0.97 | 0.99 | 17.748% | 19.351% | 1.52 | 1.59 | 10.801% | 11.447% | 0.92 | 0.94 |
| Total | 12.992% | 13.547% | 13.400% | 14.388% | 1.03 | 1.06 | 24.013% | 25.870% | 1.85 | 1.91 | 12.624% | 13.941% | 0.97 | 1.03 |

**Panel D. Omicron Period (January-June 2022). Indiana and Wisconsin**

|  | **White** | | **Black** | | | | **Hispanic** | | | | **Other** | | | |
| --- | --- | --- | --- | --- | --- | --- | --- | --- | --- | --- | --- | --- | --- | --- |
| Age | CEMP | CEMP-2019 | CEMP | CEMP-2019 | CEMP Ratio to White | CEMP-2019 Ratio to White | CEMP | CEMP-2019 | CEMP Ratio to White | CEMP-2019 Ratio to White | CEMP | CEMP-2019 | CEMP Ratio to White | CEMP-2019 Ratio to White |
| 18-39 | 13.504% | 12.701% | 10.000% | 9.211% | 0.74 | 0.73 | 28.571% | 30.189% | 2.12 | 2.38 | 25.926% | 33.333% | 1.92 | 2.62 |
| 40-49 | 12.979% | 11.790% | 15.000% | 14.537% | 1.16 | 1.23 | 27.083% | 31.707% | 2.09 | 2.69 | 20.755% | 27.500% | 1.60 | 2.33 |
| 50-59 | 14.223% | 11.834% | 11.135% | 9.369% | 0.78 | 0.79 | 17.742% | 20.183% | 1.25 | 1.71 | 13.821% | 19.540% | 0.97 | 1.65 |
| 60-69 | 11.111% | 11.537% | 15.428% | 14.956% | 1.39 | 1.30 | 25.824% | 27.647% | 2.32 | 2.40 | 12.963% | 17.500% | 1.17 | 1.52 |
| 70-79 | 10.629% | 11.111% | 11.599% | 12.546% | 1.09 | 1.13 | 27.513% | 29.379% | 2.59 | 2.64 | 15.054% | 22.222% | 1.42 | 2.00 |
| 80-89 | 8.694% | 8.140% | 13.419% | 11.732% | 1.54 | 1.44 | 15.736% | 16.489% | 1.81 | 2.03 | 6.989% | 10.000% | 0.80 | 1.23 |
| 90+ | 6.837% | 6.275% | 12.838% | 11.209% | 1.88 | 1.79 | 13.402% | 13.265% | 1.96 | 2.11 | 5.952% | 8.929% | 0.87 | 1.42 |
| 18-59 | 13.862% | 11.920% | 11.971% | 10.600% | 0.86 | 0.89 | 23.188% | 26.230% | 1.67 | 2.20 | 17.241% | 23.649% | 1.24 | 1.98 |
| 60+ | 9.312% | 9.122% | 13.417% | 12.966% | 1.44 | 1.42 | 21.504% | 22.591% | 2.31 | 2.48 | 10.841% | 15.509% | 1.16 | 1.70 |
| Total | 9.765% | 9.435% | 13.078% | 12.373% | 1.34 | 1.31 | 21.998% | 23.603% | 2.25 | 2.50 | 12.424% | 17.586% | 1.27 | 1.86 |

# Table App-10. Comparison of CEMP Ratios for Minority Groups

Table supplements text Table 2 by providing expanded set of CEMP ratios for different race/ethnic groups: Hispanic/Black; Other/Black, and Other/Hispanic. Remaining columns are same as Table 2.

**Panel A. Pre-Vaccine Period (April-December 2020). Indiana, Wisconsin, and Illinois**

|  | CEMP | | | | CEMP Ratio to White | | | CEMP Ratio to Black | | CEMP Ratio to Hispanic |
| --- | --- | --- | --- | --- | --- | --- | --- | --- | --- | --- |
| Age Bracket | White | Black | Hispanic | Other | Black | Hispanic | Other | Hispanic | Other | Other |
| 18-39 | 4.87% | 14.71% | 50.57% | 20.95% | 3.02 | 10.38 | 4.30 | 3.44 | 1.42 | 0.41 |
| 40-49 | 6.98% | 15.80% | 62.73% | 25.00% | 2.26 | 8.99 | 3.58 | 3.97 | 1.58 | 0.40 |
| 50-59 | 7.61% | 17.88% | 78.81% | 24.07% | 2.35 | 10.35 | 3.16 | 4.41 | 1.35 | 0.31 |
| 60-69 | 11.79% | 20.46% | 76.18% | 31.49% | 1.74 | 6.46 | 2.67 | 3.72 | 1.54 | 0.41 |
| 70-79 | 17.06% | 25.86% | 66.96% | 35.09% | 1.52 | 3.92 | 2.06 | 2.59 | 1.36 | 0.52 |
| 80-89 | 19.54% | 24.64% | 44.62% | 30.52% | 1.26 | 2.28 | 1.56 | 1.81 | 1.24 | 0.68 |
| 90+ | 19.20% | 23.61% | 33.69% | 28.36% | 1.23 | 1.76 | 1.48 | 1.43 | 1.20 | 0.84 |
| 18-59 | 7.18% | 16.82% | 68.69% | 23.66% | 2.34 | 9.57 | 3.30 | 4.08 | 1.41 | 0.34 |
| 60+ | 17.39% | 23.52% | 57.57% | 31.68% | 1.35 | 3.31 | 1.82 | 2.45 | 1.35 | 0.55 |
| Total | 16.31% | 22.03% | 60.42% | 30.34% | 1.35 | 3.70 | 1.86 | 2.74 | 1.38 | 0.50 |

**Panel B. Early Vaccine Period (January-June 2021). Indiana, Wisconsin, and Illinois**

|  | CEMP | | | | CEMP Ratio to White | | | CEMP Ratio to Black | | CEMP Ratio to Hispanic |
| --- | --- | --- | --- | --- | --- | --- | --- | --- | --- | --- |
| Age Bracket | White | Black | Hispanic | Other | Black | Hispanic | Other | Hispanic | Other | Other |
| 18-39 | 5.85% | 11.49% | 23.76% | 9.52% | 1.96 | 4.06 | 1.63 | 2.07 | 0.83 | 0.40 |
| 40-49 | 7.01% | 13.62% | 39.92% | 18.42% | 1.94 | 5.69 | 2.63 | 2.93 | 1.35 | 0.46 |
| 50-59 | 8.14% | 12.65% | 39.38% | 19.50% | 1.55 | 4.84 | 2.40 | 3.11 | 1.54 | 0.50 |
| 60-69 | 8.66% | 11.81% | 41.05% | 17.73% | 1.36 | 4.74 | 2.05 | 3.48 | 1.50 | 0.43 |
| 70-79 | 10.24% | 12.73% | 32.62% | 20.91% | 1.24 | 3.19 | 2.04 | 2.56 | 1.64 | 0.64 |
| 80-89 | 9.32% | 11.37% | 19.38% | 11.76% | 1.22 | 2.08 | 1.26 | 1.71 | 1.04 | 0.61 |
| 90+ | 8.06% | 9.47% | 12.83% | 11.07% | 1.18 | 1.59 | 1.37 | 1.35 | 1.17 | 0.86 |
| 18-59 | 7.63% | 12.70% | 36.49% | 17.40% | 1.66 | 4.78 | 2.28 | 2.87 | 1.37 | 0.48 |
| 60+ | 9.18% | 11.69% | 28.02% | 15.52% | 1.27 | 3.05 | 1.69 | 2.40 | 1.33 | 0.55 |
| Total | 9.01% | 11.92% | 30.18% | 15.85% | 1.32 | 3.35 | 1.76 | 2.53 | 1.33 | 0.53 |

**Panel C. Delta Period (July-December 2021). Indiana, Wisconsin, and Illinois**

|  | CEMP | | | | CEMP Ratio to White | | | CEMP Ratio to Black | | CEMP Ratio to Hispanic |
| --- | --- | --- | --- | --- | --- | --- | --- | --- | --- | --- |
| Age Bracket | White | Black | Hispanic | Other | Black | Hispanic | Other | Hispanic | Other | Other |
| 18-39 | 26.00% | 26.01% | 47.09% | 31.17% | 1.00 | 1.81 | 1.20 | 1.81 | 1.20 | 0.66 |
| 40-49 | 28.35% | 22.53% | 49.83% | 27.91% | 0.79 | 1.76 | 0.98 | 2.21 | 1.24 | 0.56 |
| 50-59 | 22.45% | 17.61% | 35.76% | 13.93% | 0.78 | 1.59 | 0.62 | 2.03 | 0.79 | 0.39 |
| 60-69 | 17.45% | 13.60% | 27.49% | 14.70% | 0.78 | 1.58 | 0.84 | 2.02 | 1.08 | 0.53 |
| 70-79 | 13.53% | 12.07% | 18.43% | 13.72% | 0.89 | 1.36 | 1.01 | 1.53 | 1.14 | 0.74 |
| 80-89 | 10.11% | 9.28% | 12.78% | 7.81% | 0.92 | 1.26 | 0.77 | 1.38 | 0.84 | 0.61 |
| 90+ | 6.83% | 7.61% | 9.25% | 6.74% | 1.11 | 1.35 | 0.99 | 1.21 | 0.88 | 0.73 |
| 18-59 | 24.13% | 20.32% | 42.23% | 20.88% | 0.84 | 1.75 | 0.87 | 2.08 | 1.03 | 0.49 |
| 60+ | 11.69% | 11.33% | 17.75% | 10.80% | 0.97 | 1.52 | 0.92 | 1.57 | 0.95 | 0.61 |
| Total | 12.99% | 13.40% | 24.01% | 12.62% | 1.03 | 1.85 | 0.97 | 1.79 | 0.94 | 0.53 |

**Panel D. Omicron Period (January-June 2022). Indiana and Wisconsin**

|  | CEMP | | | | CEMP Ratio to White | | | CEMP Ratio to Black | | CEMP Ratio to Hispanic |
| --- | --- | --- | --- | --- | --- | --- | --- | --- | --- | --- |
| Age Bracket | White | Black | Hispanic | Other | Black | Hispanic | Other | Hispanic | Other | Other |
| 18-39 | 13.50% | 10.00% | 28.57% | 25.93% | 0.74 | 2.12 | 1.92 | 2.86 | 2.59 | 0.91 |
| 40-49 | 12.98% | 15.00% | 27.08% | 20.75% | 1.16 | 2.09 | 1.60 | 1.81 | 1.38 | 0.77 |
| 50-59 | 14.22% | 11.13% | 17.74% | 13.82% | 0.78 | 1.25 | 0.97 | 1.59 | 1.24 | 0.78 |
| 60-69 | 11.11% | 15.43% | 25.82% | 12.96% | 1.39 | 2.32 | 1.17 | 1.67 | 0.84 | 0.50 |
| 70-79 | 10.63% | 11.60% | 27.51% | 15.05% | 1.09 | 2.59 | 1.42 | 2.37 | 1.30 | 0.55 |
| 80-89 | 8.69% | 13.42% | 15.74% | 6.99% | 1.54 | 1.81 | 0.80 | 1.17 | 0.52 | 0.44 |
| 90+ | 6.84% | 12.84% | 13.40% | 5.95% | 1.88 | 1.96 | 0.87 | 1.04 | 0.46 | 0.44 |
| 18-59 | 13.86% | 11.97% | 23.19% | 17.24% | 0.86 | 1.67 | 1.24 | 1.94 | 1.44 | 0.74 |
| 60+ | 9.31% | 13.42% | 21.50% | 10.84% | 1.44 | 2.31 | 1.16 | 1.60 | 0.81 | 0.50 |
| Total | 9.76% | 13.08% | 22.00% | 12.42% | 1.34 | 2.25 | 1.27 | 1.68 | 0.95 | 0.56 |

# Table App-11. Correlations between COVID-19 Mortality Rate and Non-Covid Natural Mortality Rate within Racial/Ethnic Groups

Table shows, for the indicated racial/ethnic groups and time periods, the Pearson correlation coefficient between the Covid-19 mortality rate and the Non-Covid natural mortality rate, for bins defined by state*age group*gender: 42 bins for first three periods; 28 bins for Omicron period. Age groups are same as in text Figure 1.

| Period | States included | Correlation (Covid-MR, Non-Covid NMR) | | | |
| --- | --- | --- | --- | --- | --- |
|  |  | White | Black | Hispanic | Other |
| Pre-Vaccine (April-December 2020) | IL, IN, WI | 0.98 | 0.99 | 0.92 | 0.85 |
| Early Vaccine (January-June 2021) | IL, IN, WI | 0.97 | 0.90 | 0.72 | 0.84 |
| Delta (July-December 2021) | IL, IN, WI | 0.93 | 0.92 | 0.71 | 0.77 |
| Omicron (January-June 2022) | IN, WI | 0.95 | 0.92 | 0.86 | 0.75 |

# Figure App- 1. Correlation between Non-Covid Natural Mortality and COVID-19 Mortality: Counts

Figure is similar to text Figure 1, but shows scatter plot and correlation line for *mortality counts*, instead of *rates*. **Panel A.** Scatterplot of Non-Covid natural deaths in Illinois, Indiana, and Wisconsin against COVID-19 deaths over April-December 2020 for groups defined by state*age (18-39, 40-49, 50-59, 60-64, 65-69, 70-74, 75-79, 80-84, 85-89, 90-94, 95+)*gender*race/ethnicity*SES quintile, best-fit regression line, and Pearson correlation coefficient. Plots include 840 bins (3 states*7 age groups *2 genders*4 race/ethnicities*5 SES quintiles. **Panel B**. Same for Early-Vaccine Period (1H-2020). **Panel C**. Same for Delta Period (2H-2020). **Panel D**. Similar for Omicron Period (1H 2022). Plot includes only Indiana and Wisconsin and has 560 bins (2 states*7 age groups *2 genders*4 race/ethnicities*5 SES quintiles) for 1H-2022.

**Panel A. Pre-Vaccine Period (April-December 2020)**


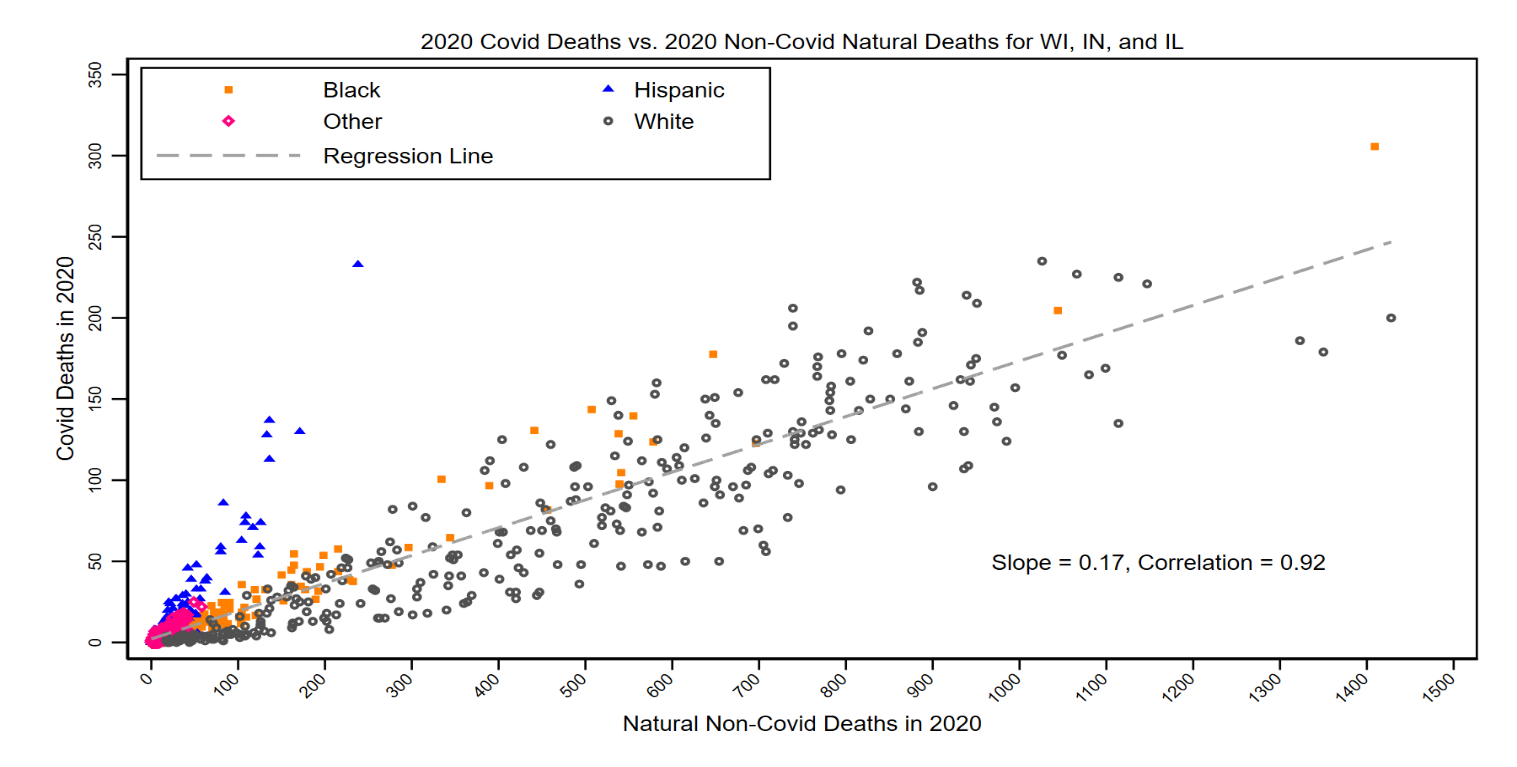


**Panel B. Early Vaccine Period (January-June 2021)**


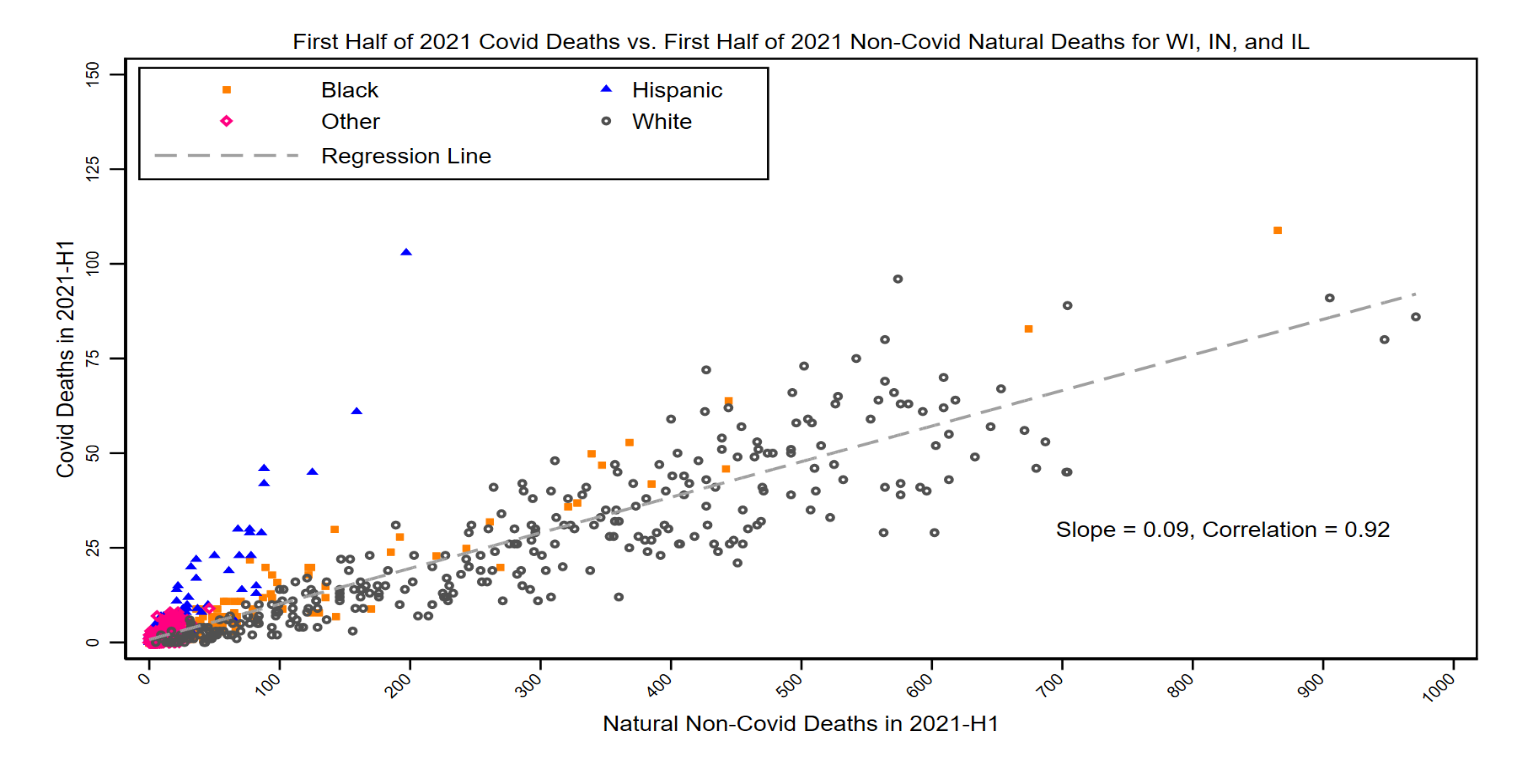


**Panel C. Delta Period (July-December 2021)**


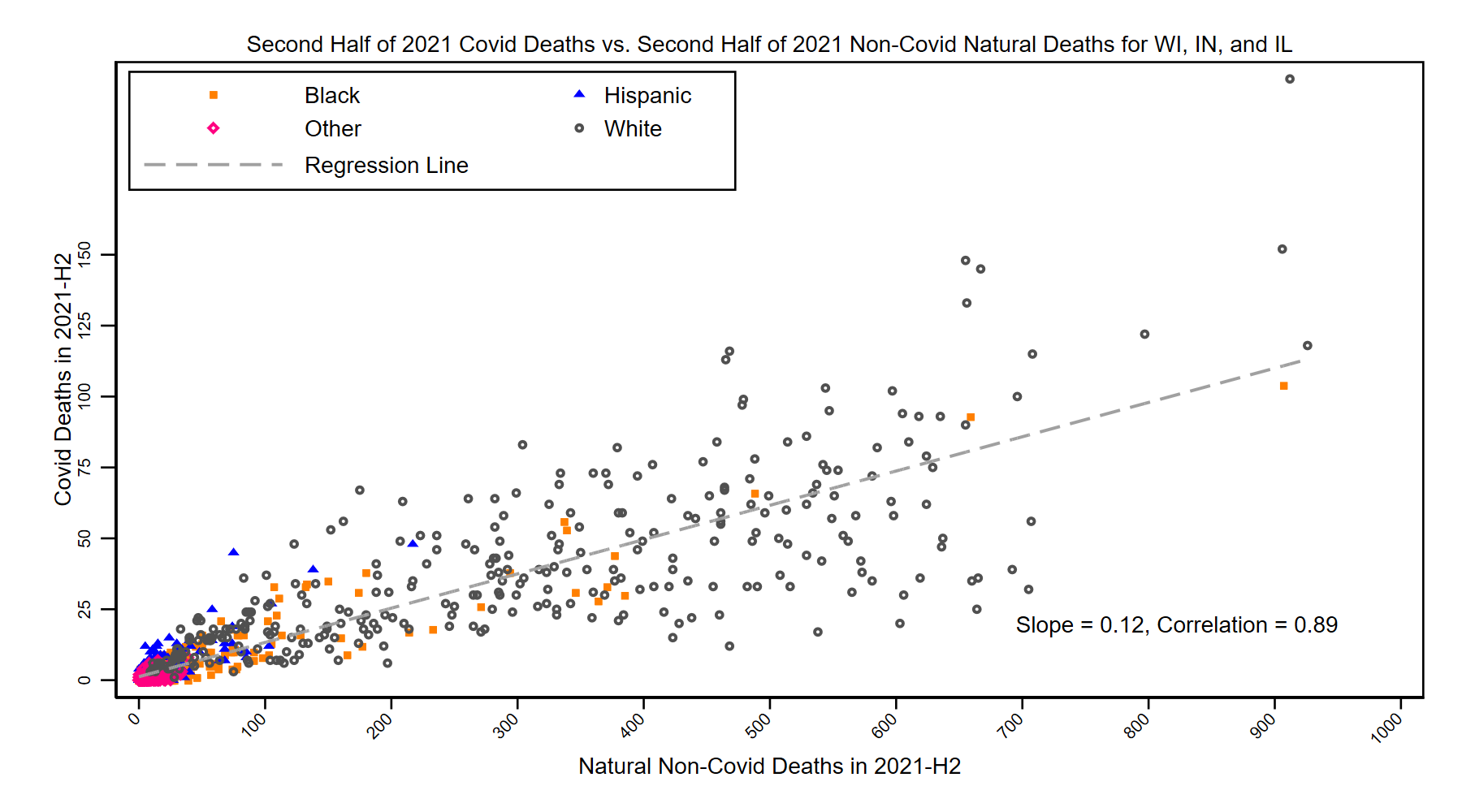


**Panel D. Omicron Period (January-June 2022)**


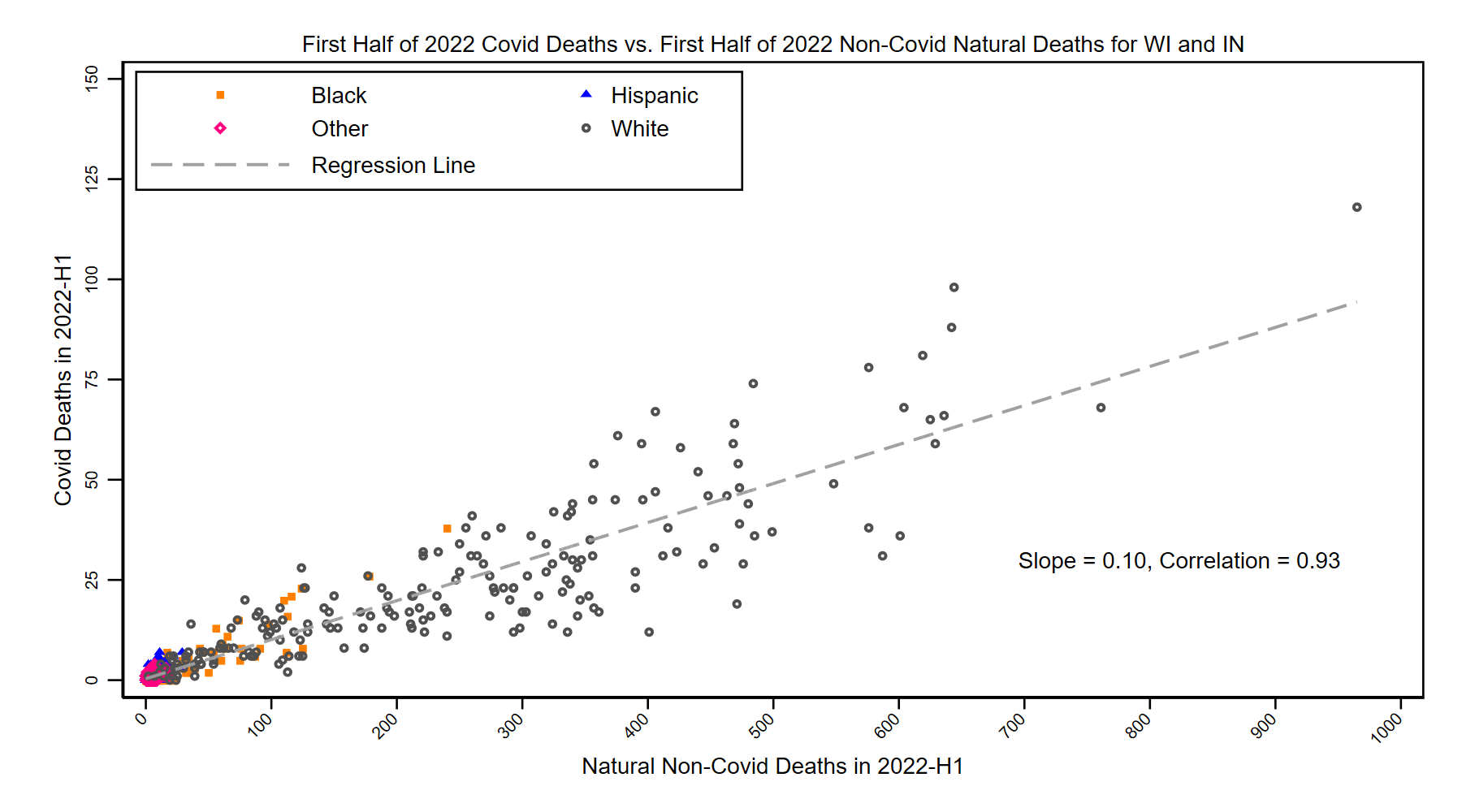


# Figure App-2. Correlation between 2019 Natural Mortality and 2020 COVID-19 Mortality

**Panel A.** Scatterplot of natural mortality deaths in Indiana over April-December 2019 against COVID-19 deaths over April-December 2020, for groups defined by age (18-39, 40-49, 50-59, 60-69, 70-79, 80-89, 90+)*gender*race/ethnicity*SES quintile, best-fit regression line, and Pearson correlation coefficient. Plot for Indiana uses zip-SES quintiles and includes 279 bins (7 age groups *2 genders*4 race/ethnicities*5 SES quintiles = 280, but one bin is empty). **Panel B**. Scatterplot for Wisconsin. Similar to Panel A, but uses county-level data instead of zip code level data for SES and race/ethnicity (zip code is not available during the pre-COVID period). Plot has 272 bins (7 age groups*2 genders*5 race/ethnicities*5 county-SES quintiles -350, but 78 bins are empty). **Panel C**. Scatterplot for Illinois. Similar to Panel A.

**Panel A. Indiana**


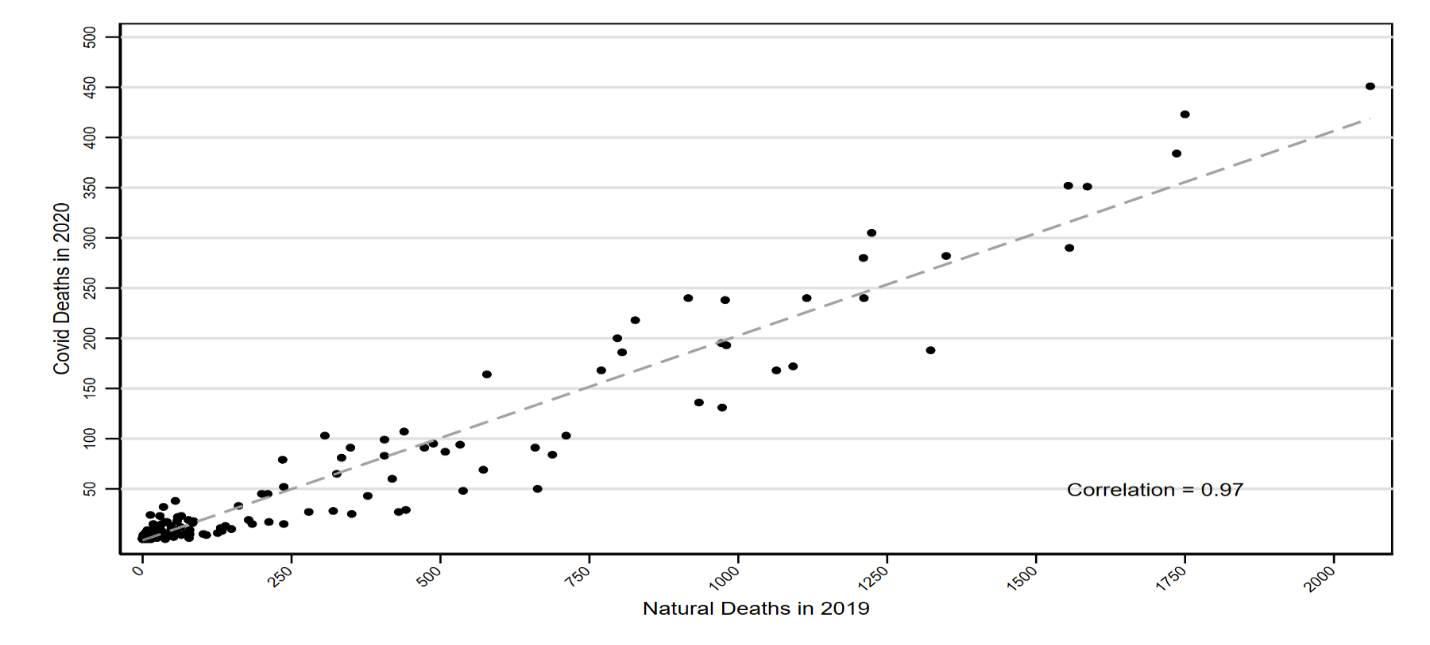
**Panel B. Wisconsin**


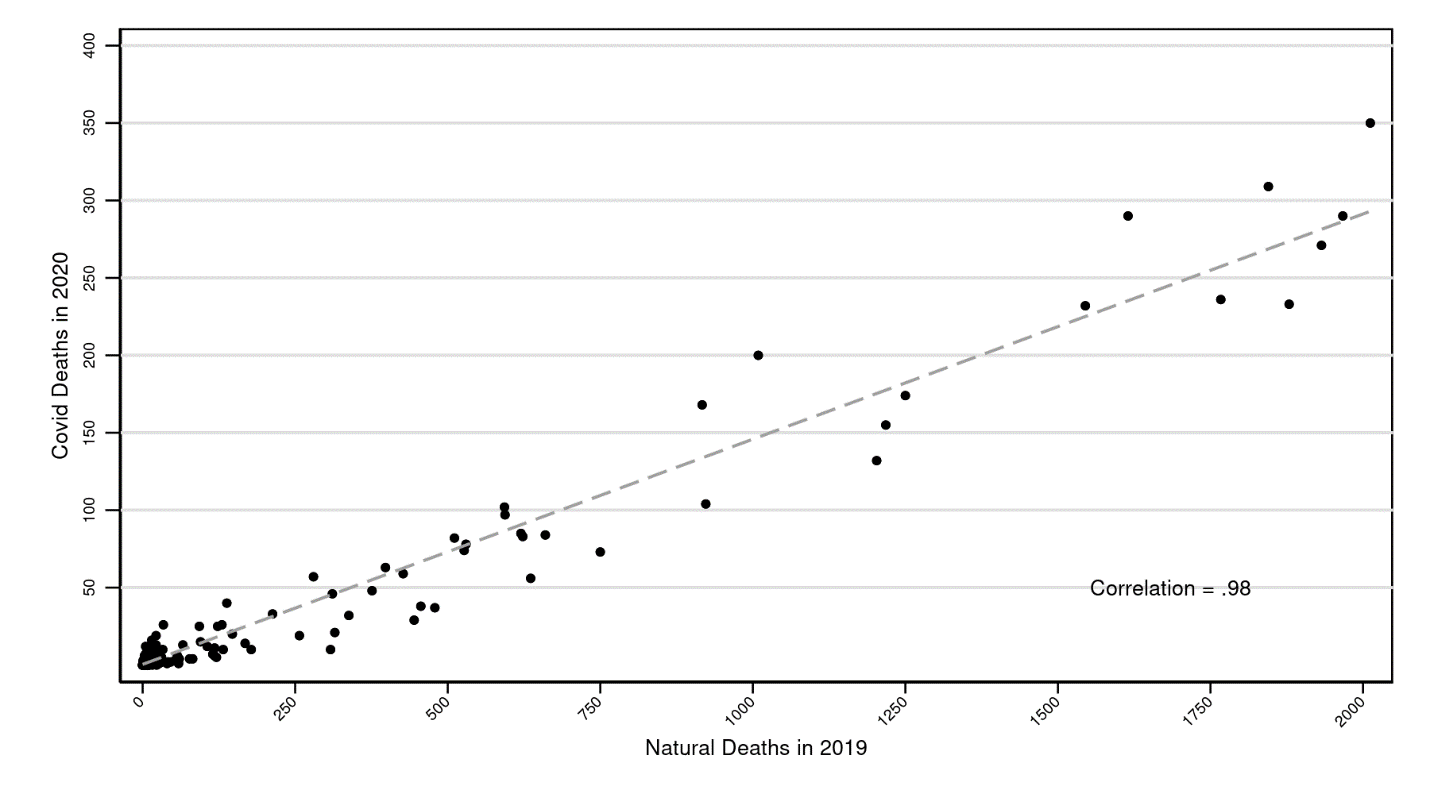


**Panel C. Illinois**

**
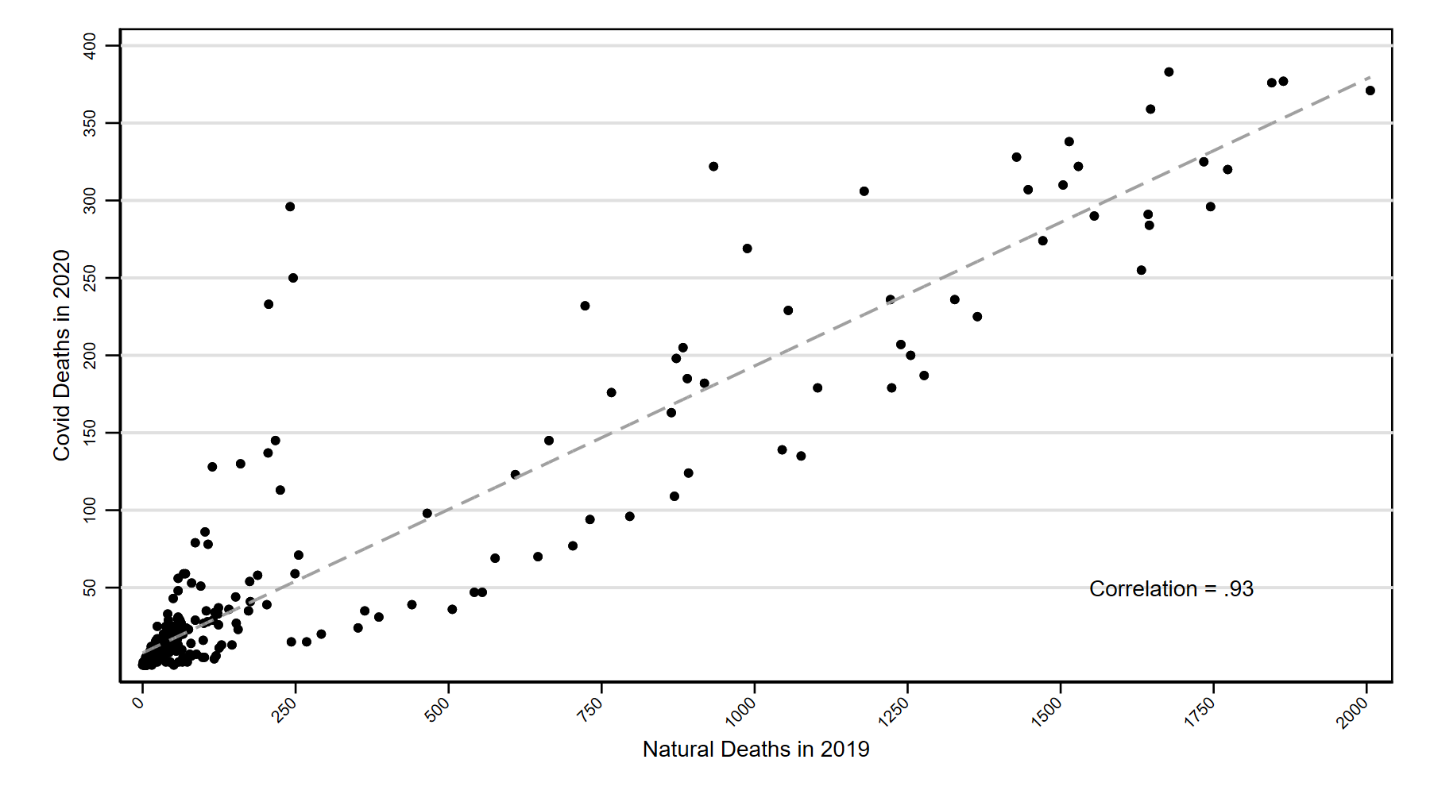
**

# Figure App-3. Actual versus Predicted Non-COVID Natural Mortality

Figure shows monthly data for natural non-COVID-19 and all natural deaths, for Indiana (**Panel A**) and Wisconsin (**Panel B**) for January 2017 – June 2022. **Panel C**. Similar, except for Illinois, and graph ends in December 2021. For the pandemic period starting March 2020, we show both actual and predicted natural non-COVID deaths. Predicted deaths are based on linear extrapolation from 2017-2019 to the same calendar month during the pandemic period. Natural deaths (including COVID-19 deaths) are shown as solid red line; this shows two large COVID-related peaks in late 2020 and late-2021-early 2022. Natural non-COVID-19 deaths (all natural deaths minus COVID-19 deaths) are shown as solid blue line. Predicted natural non-COVID deaths are shown as dashed green line.

**Panel A. Indiana**


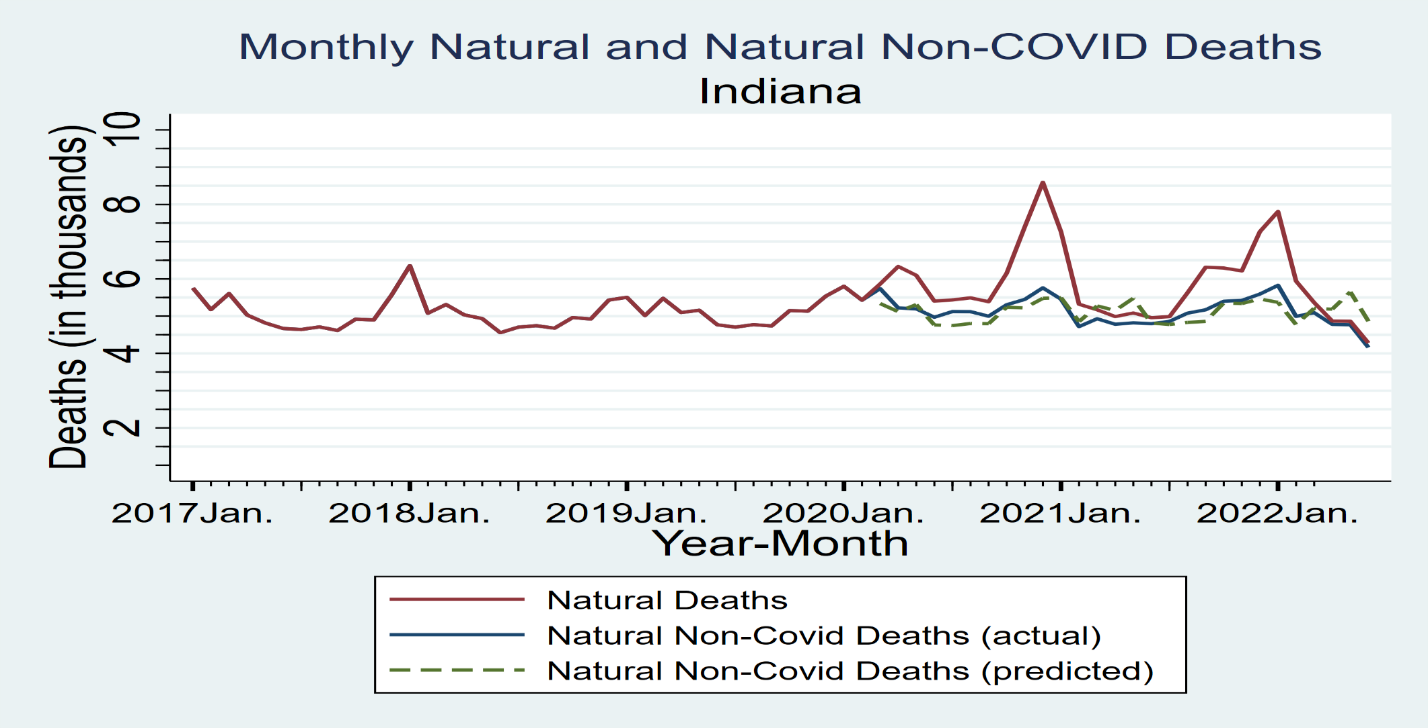


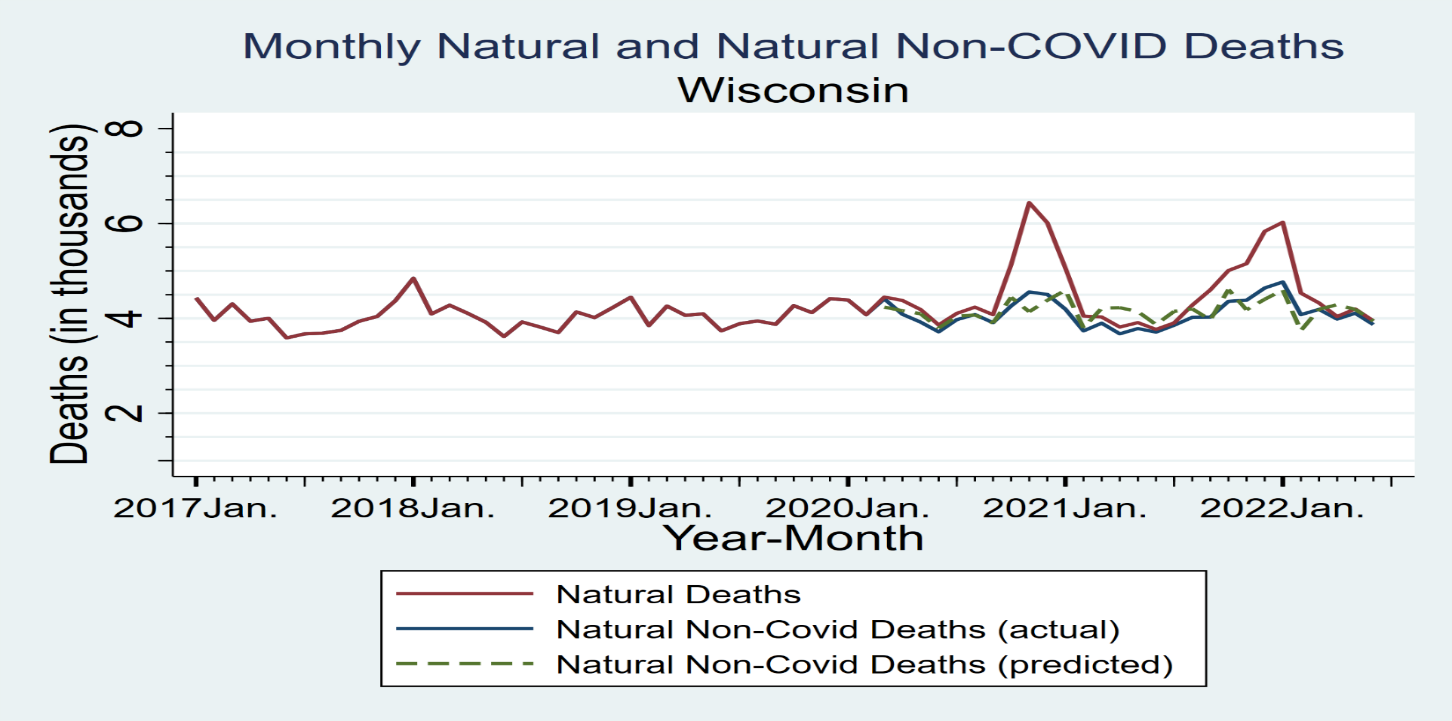
**Panel B. Wisconsin**

**Panel C. Illinois**


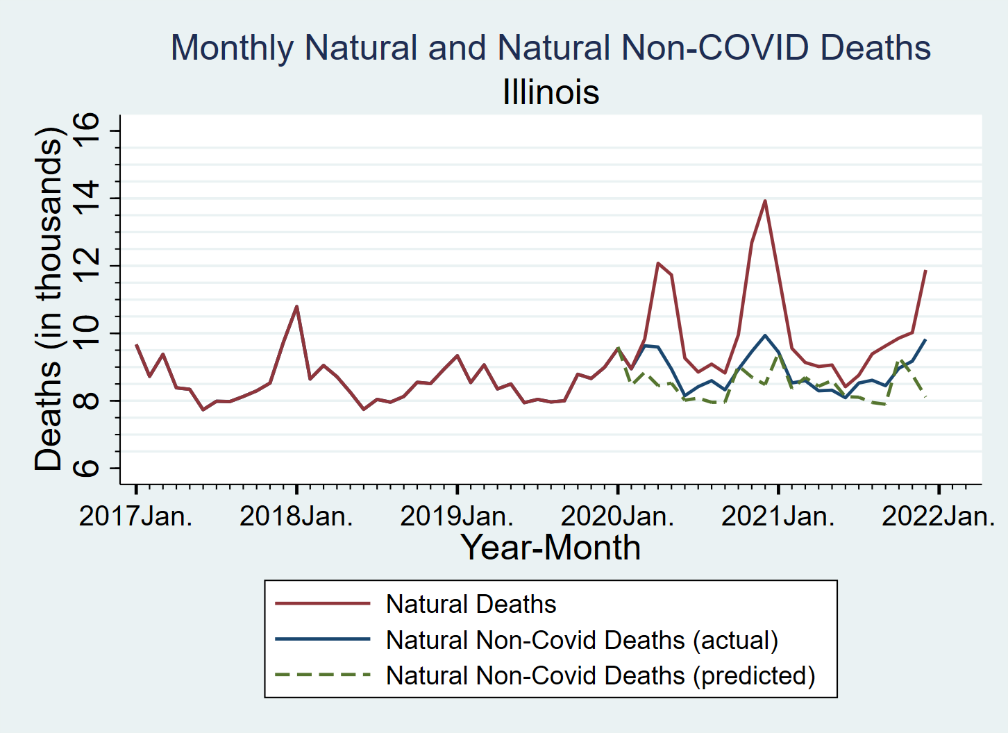


# Figure App-4. Confidence Intervals for Actual vs. Predicted Non-Covid-NMRs

Figure shows monthly data for natural non-COVID-19, for Indiana (**Panel A**) and Wisconsin (**Panel B**) for January 2020 – June 2022. **Panel C**. Similar, except for Illinois; graph ends in December 2021. For the pandemic period starting March 2020, we show both actual and predicted natural non-COVID deaths with confidence intervals around the predicted values. Predicted deaths are based on linear extrapolation from 2017-2019 to the same calendar month during the pandemic period. Natural non-COVID-19 deaths are shown as solid blue line. Predicted natural non-COVID deaths are shown as dashed green line. Confidence intervals on predicted values are shown as dotted lines.

**Panel A. Indiana**


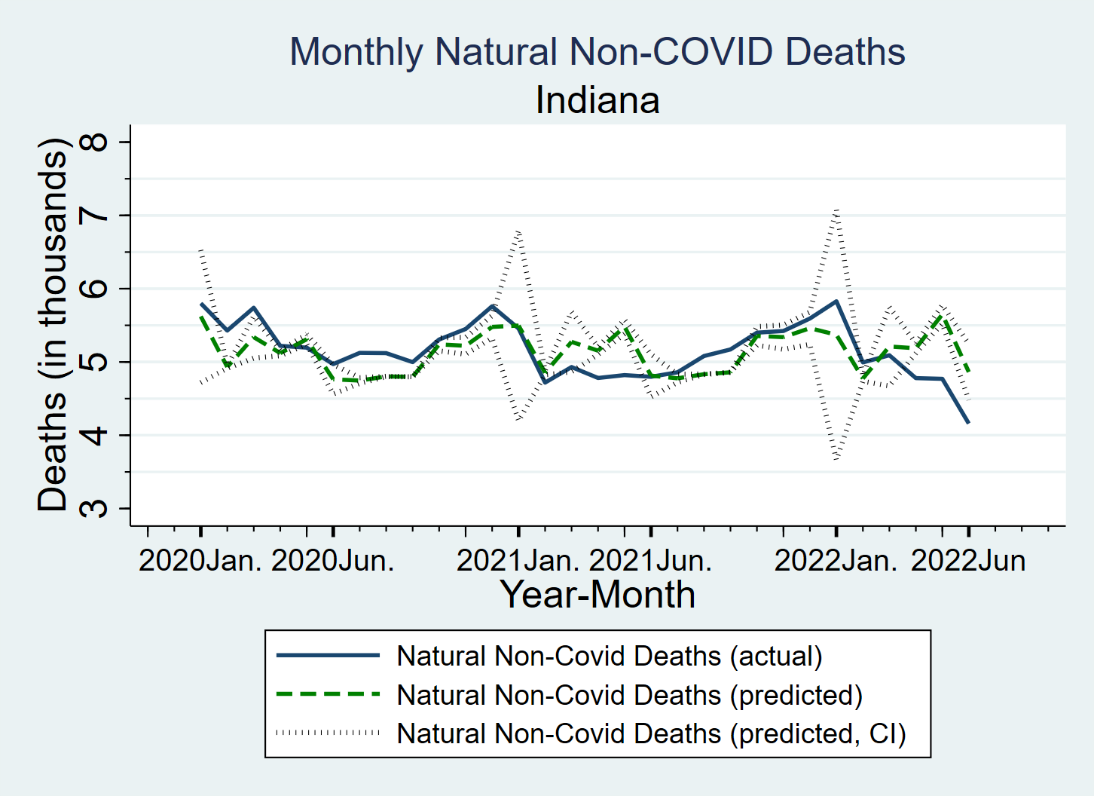


**Panel B. Wisconsin**


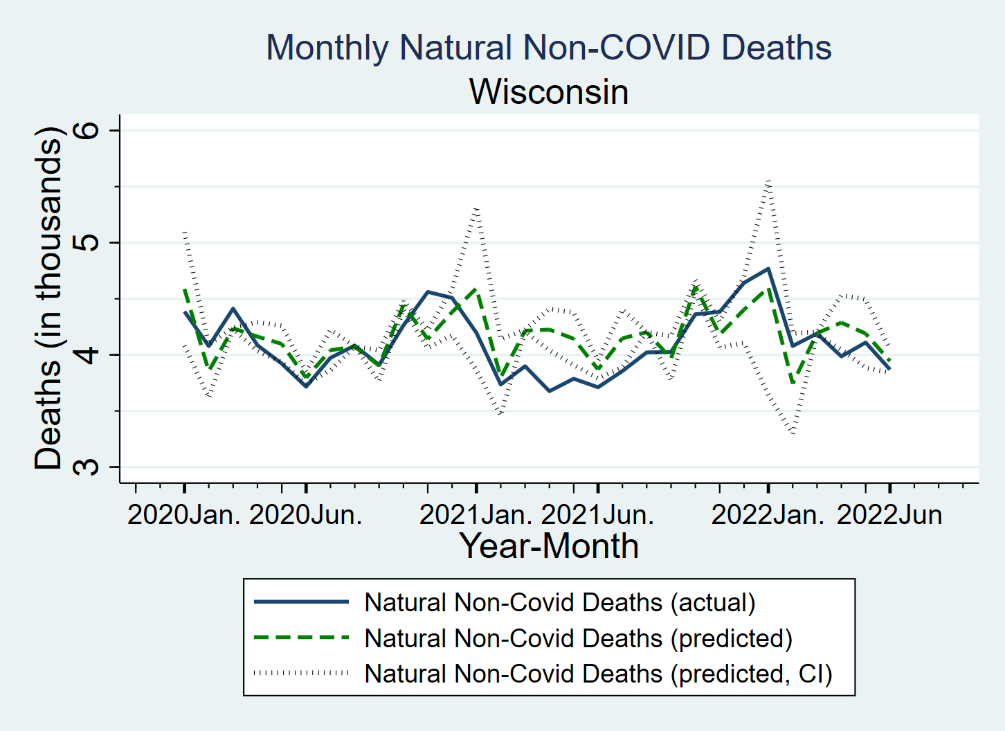


**Panel C. Illinois**


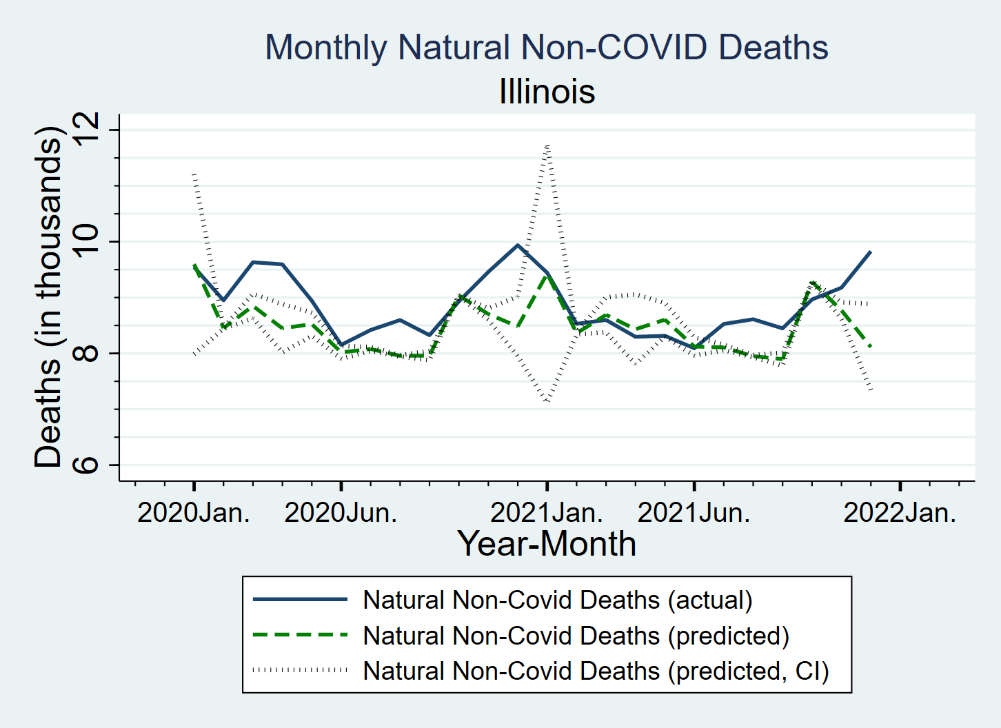


# Figure App-5. Evolution of Natural Mortality Rates for Hispanics versus Whites

Figure shows, for selected years from 2010 through 2020, national data for the ratio of Hispanic to White natural mortality for the indicated age groups. For 2020, comparison is for non-COVID natural mortality. Higher ratios (although still less than 1.00) indicate lower Hispanic mortality advantage.

1. Source: **[*url**] to come. [↑](#footnote-ref-1)
2. The 2020 Census provides slightly smaller estimates for Milwaukee (difference = 1.2%), but without the detailed age breakdowns available from ACS. We chose to use the ACS because it had more details. [↑](#footnote-ref-2)
