# Supplementary material for: Evidence on COVID-19 Mortality and Disparities Using a Novel Measure, COVID excess mortality percentage: Evidence from Indiana, Wisconsin, and Illinois
Source: PLoS One. 2024 Jan 31;19(1):e0295936. doi: 10.1371/journal.pone.0295936 (PMC10829977; doi:10.1371/journal.pone.0295936)
Supplement: Attachment — Submitted filename: 2023-09-11-PLos-One-referees-replies-bb.docx [file pone.0295936.s002.docx]

**From:** [em.pone.0.85227f.81751360@editorialmanager.com](mailto:em.pone.0.85227f.81751360@editorialmanager.com) <[em.pone.0.85227f.81751360@editorialmanager.com](mailto:em.pone.0.85227f.81751360@editorialmanager.com)> on behalf of PLOS ONE <[em@editorialmanager.com](mailto:em@editorialmanager.com)>
**Date:** Friday, August 4, 2023 at 11:10 AM
**To:** Ye Yuan <[andyyuan@law.northwestern.edu](mailto:andyyuan@law.northwestern.edu)>
**Subject:** PLOS ONE Decision: Revision required [PONE-D-23-10973] - [EMID:9b2786dd4645695c]

PONE-D-23-10973

Evidence on COVID-19 Mortality and Disparities Using a Novel Measure, COVID Excess Mortality Percentage: Evidence from Indiana, Wisconsin, and Illinois

PLOS ONE

Dear Dr. Yuan,

Thank you for submitting your manuscript to PLOS ONE. After careful consideration, we feel that it has merit but does not fully meet PLOS ONE’s publication criteria as it currently stands. Therefore, we invite you to submit a revised version of the manuscript that addresses the points raised during the review process.

The reviewers reached a consensus of major revision. After reading the comments and the submitted manuscript, the editor agrees with such consensus and believes that reviewers' comments are crucial for enhancing the quality of the manuscript to reach the publication standard of PLOS ONE. Please revise your paper thoroughly according to the comments from the two reviewers and provide an itemized response letter.

Please submit your revised manuscript by Sep 18 2023 11:59PM. If you will need more time than this to complete your revisions, please reply to this message or contact the journal office at [plosone@plos.org](mailto:plosone@plos.org). When you're ready to submit your revision, log on to [https://www.editorialmanager.com/pone/](https://urldefense.com/v3/__https:/www.editorialmanager.com/pone/__;!!H8mHWRdzp34!4raga_I6Al0HFvMXRCsecm-ZP2Rh3txMDAE042nBbuPILvfIqEOvS94w7oO43zea0oOpMjfQeYP-Cp1VKaMuEwnJl7k$) and select the 'Submissions Needing Revision' folder to locate your manuscript file.

Please include the following items when submitting your revised manuscript:

- A rebuttal letter that responds to each point raised by the academic editor and reviewer(s). You should upload this letter as a separate file labeled 'Response to Reviewers'.
- A marked-up copy of your manuscript that highlights changes made to the original version. You should upload this as a separate file labeled 'Revised Manuscript with Track Changes'.
- An unmarked version of your revised paper without tracked changes. You should upload this as a separate file labeled 'Manuscript'.

If you would like to make changes to your financial disclosure, please include your updated statement in your cover letter. Guidelines for resubmitting your figure files are available below the reviewer comments at the end of this letter.

If applicable, we recommend that you deposit your laboratory protocols in protocols.io to enhance the reproducibility of your results. Protocols.io assigns your protocol its own identifier (DOI) so that it can be cited independently in the future. For instructions see: [https://journals.plos.org/plosone/s/submission-guidelines#loc-laboratory-protocols](https://urldefense.com/v3/__https:/journals.plos.org/plosone/s/submission-guidelines*loc-laboratory-protocols__;Iw!!Dq0X2DkFhyF93HkjWTBQKhk!U-p-oUp8GBuMyOE-RsRlz20UOEYuYYUfwDVjN4RDSC6ZaVAO_p4nEY4tHGxjtO9IChigAJcoNwN25EF0f6_YDtXVmEk$). Additionally, PLOS ONE offers an option for publishing peer-reviewed Lab Protocol articles, which describe protocols hosted on protocols.io. Read more information on sharing protocols at [https://plos.org/protocols?utm_medium=editorial-email&utm_source=authorletters&utm_campaign=protocols](https://urldefense.com/v3/__https:/plos.org/protocols?utm_medium=editorial-email&utm_source=authorletters&utm_campaign=protocols__;!!Dq0X2DkFhyF93HkjWTBQKhk!U-p-oUp8GBuMyOE-RsRlz20UOEYuYYUfwDVjN4RDSC6ZaVAO_p4nEY4tHGxjtO9IChigAJcoNwN25EF0f6_YjcJ9fOg$).

We look forward to receiving your revised manuscript.

Kind regards,

Chenfeng Xiong

Academic Editor, PLOS ONE

Journal Requirements:

When submitting your revision, we need you to address these additional requirements.

1. Please ensure that your manuscript meets PLOS ONE's style requirements, including those for file naming. The PLOS ONE style templates can be found at

[https://journals.plos.org/plosone/s/file?id=wjVg/PLOSOne_formatting_sample_main_body.pdf](https://urldefense.com/v3/__https:/journals.plos.org/plosone/s/file?id=wjVg*PLOSOne_formatting_sample_main_body.pdf__;Lw!!Dq0X2DkFhyF93HkjWTBQKhk!U-p-oUp8GBuMyOE-RsRlz20UOEYuYYUfwDVjN4RDSC6ZaVAO_p4nEY4tHGxjtO9IChigAJcoNwN25EF0f6_YYpl-SwQ$) and

[https://journals.plos.org/plosone/s/file?id=ba62/PLOSOne_formatting_sample_title_authors_affiliations.pdf](https://urldefense.com/v3/__https:/journals.plos.org/plosone/s/file?id=ba62*PLOSOne_formatting_sample_title_authors_affiliations.pdf__;Lw!!Dq0X2DkFhyF93HkjWTBQKhk!U-p-oUp8GBuMyOE-RsRlz20UOEYuYYUfwDVjN4RDSC6ZaVAO_p4nEY4tHGxjtO9IChigAJcoNwN25EF0f6_YvRPIzuo$)

2. Thank you for stating in your Funding Statement:

"Research reported in this publication was supported by the National Center for Advancing Translational Sciences of the National Institutes of Health under Award Number UL1TR001436 to the Medical College of Wisconsin Clinical and Translational Science Institute of Southeast Wisconsin.  The content is solely the responsibility of the authors and does not necessarily represent the official views of the NIH."

Please provide an amended statement that declares *all* the funding or sources of support (whether external or internal to your organization) received during this study, as detailed online in our guide for authors at [http://journals.plos.org/plosone/s/submit-now.](https://urldefense.com/v3/__http:/journals.plos.org/plosone/s/submit-now.__;!!Dq0X2DkFhyF93HkjWTBQKhk!U-p-oUp8GBuMyOE-RsRlz20UOEYuYYUfwDVjN4RDSC6ZaVAO_p4nEY4tHGxjtO9IChigAJcoNwN25EF0f6_Y-u9eZMw$)  Please also include the statement “There was no additional external funding received for this study.” in your updated Funding Statement.

Please include your amended Funding Statement within your cover letter. We will change the online submission form on your behalf.

**Reply**. Done; please see the cover letter for the revised text.

3. Thank you for stating the following financial disclosure:

"Research reported in this publication was supported by the National Center for Advancing Translational Sciences of the National Institutes of Health under Award Number UL1TR001436 to the Medical College of Wisconsin Clinical and Translational Science Institute of Southeast Wisconsin.  The content is solely the responsibility of the authors and does not necessarily represent the official views of the NIH."

Please state what role the funders took in the study. If the funders had no role, please state: ""The funders had no role in study design, data collection and analysis, decision to publish, or preparation of the manuscript.""

If this statement is not correct you must amend it as needed.

Please include this amended Role of Funder statement in your cover letter; we will change the online submission form on your behalf.

**Reply**. Done; please see the cover letter for the revised text.

4. In your Data Availability statement, you have not specified where the minimal data set underlying the results described in your manuscript can be found. PLOS defines a study's minimal data set as the underlying data used to reach the conclusions drawn in the manuscript and any additional data required to replicate the reported study findings in their entirety. All PLOS journals require that the minimal data set be made fully available. For more information about our data policy, please see [http://journals.plos.org/plosone/s/data-availability](https://urldefense.com/v3/__http:/journals.plos.org/plosone/s/data-availability__;!!Dq0X2DkFhyF93HkjWTBQKhk!U-p-oUp8GBuMyOE-RsRlz20UOEYuYYUfwDVjN4RDSC6ZaVAO_p4nEY4tHGxjtO9IChigAJcoNwN25EF0f6_YaAsDFA4$).

Upon re-submitting your revised manuscript, please upload your study’s minimal underlying data set as either Supporting Information files or to a stable, public repository and include the relevant URLs, DOIs, or accession numbers within your revised cover letter. For a list of acceptable repositories, please see [http://journals.plos.org/plosone/s/data-availability#loc-recommended-repositories](https://urldefense.com/v3/__http:/journals.plos.org/plosone/s/data-availability*loc-recommended-repositories__;Iw!!Dq0X2DkFhyF93HkjWTBQKhk!U-p-oUp8GBuMyOE-RsRlz20UOEYuYYUfwDVjN4RDSC6ZaVAO_p4nEY4tHGxjtO9IChigAJcoNwN25EF0f6_YiWIQbjc$). Any potentially identifying patient information must be fully anonymized.

Important: If there are ethical or legal restrictions to sharing your data publicly, please explain these restrictions in detail. Please see our guidelines for more information on what we consider unacceptable restrictions to publicly sharing data: [http://journals.plos.org/plosone/s/data-availability#loc-unacceptable-data-access-restrictions](https://urldefense.com/v3/__http:/journals.plos.org/plosone/s/data-availability*loc-unacceptable-data-access-restrictions__;Iw!!Dq0X2DkFhyF93HkjWTBQKhk!U-p-oUp8GBuMyOE-RsRlz20UOEYuYYUfwDVjN4RDSC6ZaVAO_p4nEY4tHGxjtO9IChigAJcoNwN25EF0f6_YICidYLk$). Note that it is not acceptable for the authors to be the sole named individuals responsible for ensuring data access.

**Reply**. We are not permitted to provide publicly the data on which we rely. We can, however, provide our statistical code if desired. We can also provide a link to a dataset, on Professor Black’s professional website, that includes the Graham Social Deprivation Index at zip-code level. Please advise on whether this would be helpful. Subject to that, with regard to data availability, we propose the following statement.

The Illinois, Indiana, and Wisconsin mortality data on which we rely was obtained from the respective state Departments of Health and Vital Statistics, and is covered by data use agreements which prevent us from publicly releasing it. The zip-code level Graham Social Deprivation Index, which relies on data from the publicly available American Community Survey, and zip-code level population data, is available from Professor Black’s website at Northwestern University. Go to <https://www.law.northwestern.edu/faculty/profiles/bernardblack/>; click on “datasets”. The zip-code level population data we use is from the American Community Survey. The statistical code we relied on is available from the authors on request.

We will update your Data Availability statement to reflect the information you provide in your cover letter.

**Reply**. We included the text above in our cover letter.

5. Your ethics statement should only appear in the Methods section of your manuscript. If your ethics statement is written in any section besides the Methods, please delete it from any other section.

**Reply**. Not applicable. We use only de-identified data on mortality. Thus, our study does not involve human subjects research.

**Reviewers’ Comments to the Author**

1. Is the manuscript technically sound, and do the data support the conclusions?

The manuscript must describe a technically sound piece of scientific research with data that supports the conclusions. Experiments must have been conducted rigorously, with appropriate controls, replication, and sample sizes. The conclusions must be drawn appropriately based on the data presented.

Reviewer #1: Yes

Reviewer #2: Partly

2. Has the statistical analysis been performed appropriately and rigorously?

Reviewer #1: No

Reviewer #2: Yes

3. Have the authors made all data underlying the findings in their manuscript fully available?

The [PLOS Data policy](https://urldefense.com/v3/__http:/www.plosone.org/static/policies.action*sharing__;Iw!!Dq0X2DkFhyF93HkjWTBQKhk!U-p-oUp8GBuMyOE-RsRlz20UOEYuYYUfwDVjN4RDSC6ZaVAO_p4nEY4tHGxjtO9IChigAJcoNwN25EF0f6_YDhtXlxc$) requires authors to make all data underlying the findings described in their manuscript fully available without restriction, with rare exception (please refer to the Data Availability Statement in the manuscript PDF file). The data should be provided as part of the manuscript or its supporting information, or deposited to a public repository. For example, in addition to summary statistics, the data points behind means, medians and variance measures should be available. If there are restrictions on publicly sharing data—e.g. participant privacy or use of data from a third party—those must be specified.

Reviewer #1: No

Reviewer #2: No

4. Is the manuscript presented in an intelligible fashion and written in standard English?

PLOS ONE does not copyedit accepted manuscripts, so the language in submitted articles must be clear, correct, and unambiguous. Any typographical or grammatical errors should be corrected at revision, so please note any specific errors here.

Reviewer #1: Yes

Reviewer #2: Yes

**5. Review Comments to the Author**

Please use the space provided to explain your answers to the questions above. You may also include additional comments for the author, including concerns about dual publication, research ethics, or publication ethics. (Please upload your review as an attachment if it exceeds 20,000 characters)

**Reviewer #1:** This paper examines a new measure—the COVID Excess Mortality Percentage or CEMP—as an alternative way to understand disparities in COVID-19 throughout the course of the pandemic in three midwestern states. The separation into different pandemic periods (pre-vaccine, early vaccine, Delta, Omicron) is a strength of this paper and shows how disparities have evolved over time.

My primary concern is with the use of contemporary non-COVID-19 mortality rates in the CEMP rather than pre-pandemic mortality. The introduction notes that this novel measure takes into account background risk and was validated by comparing 2019 natural mortality to 2020 COVID-19 mortality. That seems very reasonable, but the use of contemporaneous COVID-19 and other natural mortality in the present study does not. Prior research has shown that COVID-19 is a competing risk of death with several natural causes, and may be so differentially by population. The paper would be more convincing if pre-pandemic mortality were used so as not to contaminate the non-COVID mortality rates used for background risk.

**Reply**. Thank you for this suggestion. Given the very high correlation between 2019 natural mortality and 2020 non-COVID natural mortality, within age group*race/ethnicity buckets (Figure App-2), we would expect, and in the revised draft confirm, that our results are similar using either contemporaneous or 2019 natural mortality in the CEMP denominator. For this project, we can see advantages and disadvantages to using either denominator. An advantage of using the 2019 denominator, as you note, is that pre-Covid data are not affected by COVID as a competing risk. A disadvantage is that the 2019 data do not reflect changes over time in population, or in non-COVID sources of mortality. An important benefit of using contemporaneous mortality from other natural causes is that this approach permits the multivariate decomposition and measurement of confidence intervals that we provide in Table 3.

Your speculation below about a secular decline in the Hispanic life expectancy advantage, which we confirm (see below), implies that using a denominator based on 2019 mortality will cause upward bias in CEMP for Hispanics, relative to a contemporaneous denominator by suppressing the denominator.

A further consideration for which denominator to use is that in other COVID-19 projects, we studying vaccine effectiveness; for which the contemporaneous denominator is the only one we can use, because we need to observe who is vaccinated, which is not known until vaccination starts in 2021. See Atanasov et al. (2023a, 2023b).

Given these considerations, the modest differences between the two approaches, and the value of using a consistent definition across related projects, we chose to keep the current definition in the text. However, we added two new tables to the Appendix, and discuss the differences in the text. New Table App-8 reproduces text Table 2 but uses natural mortality in 2019 as the denominator. CEMP levels are typically a bit higher, consistent with modest undercounting of COVID deaths. We had already reported modest undercounting in Illinois, so these differences are expected. New Table App-9 directly compares minority/White CEMP ratios from the two approaches and shows that these ratios are similar with either denominator.

That concern aside, the paper needs to better motivate and provide intuition about how to interpret the CEMP measure. I do not understand the “excess” in COVID Excess Mortality Percentage. Because of the way it is defined, it’s not clear what this in excess of.

**Reply**. We rephrased for clarity. The word “excess” was meant to reflect that COVID deaths in 2020-2022 are treated as being in addition to (in excess of) natural mortality from other causes. For example, a CEMP value of 20% implies that mortality from natural causes increased by 20% due to COVID-19 deaths, relative to the expected level without COVID. This involves the simplifying assumption that the COVID-19 decedents would not have died from other natural causes. As we explain in the revised limitations section, this cannot be entirely true – COVID-19 infection surely killed some number of people, especially the old and frail, who would have died soon from other natural causes.

While the NMR takes into account background health to some extent, it does not fully capture it.

**Reply**. We agree. The text is explicit, including a paragraph in the limitations section, that the non-Covid natural mortality rate is an imperfect proxy for background health.

For example, several scholars have questioned whether the Hispanic paradox can persist given rising diabetes and obesity rates. In the absence of the pandemic, these underlying risk factors may have taken many years to manifest in increased mortality in the Hispanic population, but were clearly associated with increased risk of COVID-19. The fact that NMR, and thus CEMP, cannot fully capture underlying health should be discussed more in the limitations section.

**Reply**. We were able to assess the possibility that the Hispanic mortality advantage was declining over time with a different dataset, which we had previously obtained from the National Center for Health Statistics, that provides national mortality rates by race/ethnicity, although with geography limited to the county level and with a longer delay before results are available. In the figure below, we show relative national mortality rates by age group for Hispanics and for non-Hispanic Whites in selected years from 2010 through 2020 (for 2020, we use non-COVID natural mortality).

As the figure shows, the Hispanic advantage in natural mortality is slowly but steadily declining over time. The change in mortality rates is slow, so should not strongly affect our results. The decline in the Hispanic advantage, by itself, would imply that Hispanic/White CEMP ratios, which use 2020 non-COVID natural mortality rates in the denominator, should be lower than Hispanic/White CEMP-2019 ratios, which use 2019 natural mortality rates in the denominator. We in fact find that Hispanic/White CEMP ratios similar to Hispanic/White CEMP-2019 ratios, suggesting that other differences between 2019 and 2020 non-COVID mortality rates offset any effects from the slow decline in magnitude of the Hispanic paradox.

We strengthened discussion of the limitations of CEMP in the limitations section, as you suggested. We mention the declining Hispanic advantage in the revised text, and discuss this new finding in more detail in the Appendix.

We have also conducted a careful literature search, and could not find any study of all-cause natural mortality that compares Hispanic to White mortality rates over time; only narrower studies that do so for particular causes of death, with mixed results. Thus, you have given us an insight that will lead to a separate project, now underway, that will document the declining Hispanic advantage, and speculate about its causes. Thank you!

The methods section mentions that immune-compromised individuals were excluded. What is the rationale for their exclusion? These individuals would certainly have **[*something missing here]**

**Reply**. We had dropped the immune compromised, because this was how we handled the immune-compromised when studying vaccine effectiveness in separate work. We agree that there is no good reason to do so for this study, and have revised Table 3 to use the full sample. The coefficients barely change; most do not change to the two decimal places that we report.

The use of “COVID-PFR” throughout the manuscript was confusing as there is standard demographic terminology “COVID-19 mortality rate” that is commonly used in the literature.

**Reply**. Done. We use COVID-19 MR as the abbreviation.

Figure 1 typo: the title says 2019 NMR but the axis labels show contemporaneous NMR rates.

**Reply**. Fixed.

Why do the scatterplots display absolute numbers of deaths rather than population rates? This makes it hard to see racial/ethnic disparities because minorities are all clumped together near 0 because of low population size.

**Reply**. In the revised draft, we follow your suggested approach in the text, and move the current figures to the Appendix. One limitation of the revised approach (and the reason for our approach in the prior draft) is that we have to rely on population estimates from a different data source (American Community Survey, ACS) than our relying entirely on our own mortality data. Population estimates are available from the ACS for subgroups defined by state*age range*gender* race/ethnicity, but not for the further subdivision into zip code and thus into zip-SES quintiles, which is available when we rely solely on counts from the mortality data. Thus, in the text, we report mortality rate datapoints within state*age range*gender* race/ethnicity groups. The correlations remain very strong, at r = 0.90 for 2020, 0.84 for 1H 2021, 0.82 for 2H 2021, and 0.82 for 1H 2022. We moved the prior results, relying on counts rather than rates, to the Appendix.

The text references PFR ratios when discussing Table 2, but these are not displayed so it is hard to follow.

**Reply.** We revised the column headings in Table 2 to be “COVID MR,” following your earlier comment. This should make it clearer what ratios we are referring to.

**Reviewer #2:**  The authors studied COVID-19 mortality and disparities in Indiana, Wisconsin, and Illinois. They studied that mortality risk and racial/ethnic disparities in COVID-19 mortality and how both vary with age, gender, and time period. This manuscript uses a novel measure called COVID Excess Mortality Percentage (CEMP) to inform COVID-19 mortality rates and racial/ethnic disparities. CEMP is defined as the COVID-19 mortality rate divided by the non-COVID natural mortality rate, converted as a percentage. By studying the CEMP along with the COVID-19 population fatality rate (COVID-PFR), the authors are able to separate the effects of background health from Covid-specific factors affecting COVID-19 mortality. The authors concluded that CEMP levels for racial and ethnic minority groups, especially for Hispanics, can be very high relative to White levels. Disparities were generally lower after age 60 and declined over the sample period. Socio-economic status and education contribute very little to these disparities.

Generally, I thought that the manuscript was well-written (with a few minor typos) and very completed and detailed in their description, methodology, analysis, discussion, and limitations. I have a few questions regarding the manuscript that I hope that the authors could clarify either through the review process or by updating their paper.

1. Why were minority ethnic groups only compared to the white ethnic group? Why not perform an analysis between different ethnic groups as well?

**Reply**. We added an analysis of Hispanic/Black, Other/Black, and Hispanic/Other to a new table in the Appendix, and refer to this table in the text. There was no room to add additional columns in the text.

2. I do not think that saying that the CEMP denominator strongly predicts COVID-19 mortality is valid. You can say that they are strongly correlated, but not necessarily a prediction factor, at least from my point of view. What makes this a prediction? What are some explanations for this correlation/prediction?

**Reply**. We revised to say they are correlated or, where appropriate, “strongly correlated.” For us, the term “predict” was not intended to imply causation. Instead, as we discuss in the revised draft, both the high correlation and the predictive power arise because natural non-COVID mortality rates are strongly correlated with underlying health, and underlying health is strongly correlated with risk of COVID death. For both correlations, the causal arrow presumably runs from underlying health to both COVID-19 mortality and non-COVID natural mortality.

3. For the correlation between natural mortality and COVID-19 mortality, the data points in the higher values of natural non-Covid deaths are more spread out than near the origin. This potentially could indicate that this model is not a good predictor for high mortality (since the confidence interval will need to be wider).

**Reply**. In the revised draft, we report correlation graphs for mortality rates, instead of counts. We moved the graphs with counts to the Appendix.

4. Why was the correlation between natural mortality and COVID-19 mortality done for the entire data set? Was it done for each ethnic group as well?

**Reply**. In the revised draft, we added a table with correlations within ethnic groups to the Appendix and refer to it in the text. The within-group correlations are also strong.

5. Additionally, the authors mentioned that all ethnic minority groups had the majority of their data points above the regression line. All this means is that their CEMP is higher than White (which make up most of the data points). What is the purpose of this figure as this can be calculated numerically?

**Reply**. We agree that CEMP > 1 will lead to a data point in Figure 1 above the regression line. We believe that there is value in a graphical look at the data, disaggregated by age group*gender*race/ethnicity, which Table 2 does not provide.

Some typos that I caught while reading:

**General reply**. Thank you for taking the time to point these out to us.

1. Page 7: space required after "=" for Non-COVID NMR

**Reply**. Fixed (or actually, not found, so we probably caught it ourselves)

2. Page 7: If you are using equation numbers, please use different equation numbers for different equations (you have equation #1 appearing twice). Additionally, there is no purpose of including equation numbers if you will not be referencing the equation in your text.

**Reply**. We removed the equation numbers.

3. Page 8: Should be "Other =" instead of "Other -"

**Reply**. Fixed. You have good eyes!

4. Page 8, second-last paragraph: should be "First, Hispanics [...]", instead of "First. Hispanics [...]"

**Reply**. Fixed

5. Page 14: Last paragraph starting with "In 2020", the first second doesn't make sense.

**Reply**. Fixed

6. Page 15, first paragraph: should be "During the pre-vaccine period" instead of "During the pre-vaccine Period"

**Reply**. Fixed

7. Page 18, second paragraph: should be "COVID-PFR" instead of "CCOVID-PFR"

**Reply**. Fixed; we had caught this one.

6. PLOS authors have the option to publish the peer review history of their article ([what does this mean?](https://urldefense.com/v3/__https:/journals.plos.org/plosone/s/editorial-and-peer-review-process*loc-peer-review-history__;Iw!!Dq0X2DkFhyF93HkjWTBQKhk!U-p-oUp8GBuMyOE-RsRlz20UOEYuYYUfwDVjN4RDSC6ZaVAO_p4nEY4tHGxjtO9IChigAJcoNwN25EF0f6_Y0tdfFRo$)). If published, this will include your full peer review and any attached files. If you choose “no”, your identity will remain anonymous but your review may still be made public.

**Do you want your identity to be public for this peer review?** For information about this choice, including consent withdrawal, please see our [Privacy Policy](https://urldefense.com/v3/__https:/www.plos.org/privacy-policy__;!!Dq0X2DkFhyF93HkjWTBQKhk!U-p-oUp8GBuMyOE-RsRlz20UOEYuYYUfwDVjN4RDSC6ZaVAO_p4nEY4tHGxjtO9IChigAJcoNwN25EF0f6_YXrKGFDk$).

Reviewer #1: No

Reviewer #2: No

While revising your submission, please upload your figure files to the Preflight Analysis and Conversion Engine (PACE) digital diagnostic tool, [https://pacev2.apexcovantage.com/](https://urldefense.com/v3/__https:/pacev2.apexcovantage.com/__;!!Dq0X2DkFhyF93HkjWTBQKhk!U-p-oUp8GBuMyOE-RsRlz20UOEYuYYUfwDVjN4RDSC6ZaVAO_p4nEY4tHGxjtO9IChigAJcoNwN25EF0f6_Yd1cwDM4$). PACE helps ensure that figures meet PLOS requirements. To use PACE, you must first register as a user. Registration is free. Then, login and navigate to the UPLOAD tab, where you will find detailed instructions on how to use the tool. If you encounter any issues or have any questions when using PACE, please email PLOS at [figures@plos.org](mailto:figures@plos.org). Please note that Supporting Information files do not need this step.
