# Supplementary material for: Evidence on COVID-19 Mortality and Disparities Using a Novel Measure, COVID excess mortality percentage: Evidence from Indiana, Wisconsin, and Illinois
Source: PLoS One. 2024 Jan 31;19(1):e0295936. doi: 10.1371/journal.pone.0295936 (PMC10829977; doi:10.1371/journal.pone.0295936)
Supplement: Attachment — Submitted filename: PloS One round 2-editor referees rerply-2023-11-07-bb.docx [file pone.0295936.s003.docx]

**PloS One round 2 for CEMP and Race (with replies)**

**From:** [em.pone.0.8711b0.0a91545d@editorialmanager.com](mailto:em.pone.0.8711b0.0a91545d@editorialmanager.com) <[em.pone.0.8711b0.0a91545d@editorialmanager.com](mailto:em.pone.0.8711b0.0a91545d@editorialmanager.com)> on behalf of PLOS ONE <[em@editorialmanager.com](mailto:em@editorialmanager.com)>
**Date:** Tuesday, October 31, 2023 at 11:58 AM
**To:** Ye Yuan <[andyyuan@law.northwestern.edu](mailto:andyyuan@law.northwestern.edu)>
**Subject:** PLOS ONE Decision: Revision required [PONE-D-23-10973R1] - [EMID:eb0509c9f076f0ed]

PONE-D-23-10973R1

Evidence on COVID-19 Mortality and Disparities Using a Novel Measure, COVID Excess Mortality Percentage: Evidence from Indiana, Wisconsin, and Illinois

PLOS ONE

Dear Dr. Yuan,

Thank you for submitting your manuscript to PLOS ONE. After careful consideration, we feel that it has merit but does not fully meet PLOS ONE’s publication criteria as it currently stands. Therefore, we invite you to submit a revised version of the manuscript that addresses the points raised during the review process.

Please address the remaining comments from Reviewer 1. As this is the second round of review and only minor revision comments were received by reviewers, the editor reached a minor revision decision.

**Reply**: See below for our replies to Reviewer 1

And please finalize the paper and resubmit. It is worth noting that both Reviewer 1 and Reviewer 2 brought up seemingly editorial related comments. Please work on editing your paper thoroughly to make sure the writing is clean and accurate.

**Reply**: We responded to the minor editing comments from both reviewers.

Please submit your revised manuscript by Dec 15 2023 11:59PM. If you will need more time than this to complete your revisions, please reply to this message or contact the journal office at [plosone@plos.org](mailto:plosone@plos.org). When you're ready to submit your revision, log on to <https://www.editorialmanager.com/pone/> and select the 'Submissions Needing Revision' folder to locate your manuscript file.

Please include the following items when submitting your revised manuscript:

- A rebuttal letter that responds to each point raised by the academic editor and reviewer(s). You should upload this letter as a separate file labeled 'Response to Reviewers'.
- A marked-up copy of your manuscript that highlights changes made to the original version. You should upload this as a separate file labeled 'Revised Manuscript with Track Changes'.
- An unmarked version of your revised paper without tracked changes. You should upload this as a separate file labeled 'Manuscript'.

If you would like to make changes to your financial disclosure, please include your updated statement in your cover letter. Guidelines for resubmitting your figure files are available below the reviewer comments at the end of this letter.

If applicable, we recommend that you deposit your laboratory protocols in protocols.io to enhance the reproducibility of your results. Protocols.io assigns your protocol its own identifier (DOI) so that it can be cited independently in the future. For instructions see: [https://journals.plos.org/plosone/s/submission-guidelines#loc-laboratory-protocols](https://urldefense.com/v3/__https:/journals.plos.org/plosone/s/submission-guidelines*loc-laboratory-protocols__;Iw!!Dq0X2DkFhyF93HkjWTBQKhk!VMtgmOTuZXfOTJyfmPrRsHuzsafg6d3zyLSUXmbAnWg4pf4wmEPATag1H0BWXCQjkYpZ6q6_tILru_GSnjufXi-x9gM$). Additionally, PLOS ONE offers an option for publishing peer-reviewed Lab Protocol articles, which describe protocols hosted on protocols.io. Read more information on sharing protocols at [https://plos.org/protocols?utm_medium=editorial-email&utm_source=authorletters&utm_campaign=protocols](https://urldefense.com/v3/__https:/plos.org/protocols?utm_medium=editorial-email&utm_source=authorletters&utm_campaign=protocols__;!!Dq0X2DkFhyF93HkjWTBQKhk!VMtgmOTuZXfOTJyfmPrRsHuzsafg6d3zyLSUXmbAnWg4pf4wmEPATag1H0BWXCQjkYpZ6q6_tILru_GSnjufQKIhH48$).

We look forward to receiving your revised manuscript.

Kind regards,

Chenfeng Xiong
Academic Editor
PLOS ONE

Journal Requirements:

Please review your reference list to ensure that it is complete and correct. If you have cited papers that have been retracted, please include the rationale for doing so in the manuscript text, or remove these references and replace them with relevant current references. Any changes to the reference list should be mentioned in the rebuttal letter that accompanies your revised manuscript. If you need to cite a retracted article, indicate the article’s retracted status in the References list and also include a citation and full reference for the retraction notice.

Reviewers' comments:

Reviewer's Responses to Questions

**Comments to the Author**

1. If the authors have adequately addressed your comments raised in a previous round of review and you feel that this manuscript is now acceptable for publication, you may indicate that here to bypass the “Comments to the Author” section, enter your conflict of interest statement in the “Confidential to Editor” section, and submit your "Accept" recommendation.

Reviewer #1: (No Response)

Reviewer #2: All comments have been addressed.

2. Is the manuscript technically sound, and do the data support the conclusions?

The manuscript must describe a technically sound piece of scientific research with data that supports the conclusions. Experiments must have been conducted rigorously, with appropriate controls, replication, and sample sizes. The conclusions must be drawn appropriately based on the data presented.

Reviewer #1: Yes

Reviewer #2: Yes

3. Has the statistical analysis been performed appropriately and rigorously?

Reviewer #1: Yes

Reviewer #2: Yes

4. Have the authors made all data underlying the findings in their manuscript fully available?

The [PLOS Data policy](https://urldefense.com/v3/__http:/www.plosone.org/static/policies.action*sharing__;Iw!!Dq0X2DkFhyF93HkjWTBQKhk!VMtgmOTuZXfOTJyfmPrRsHuzsafg6d3zyLSUXmbAnWg4pf4wmEPATag1H0BWXCQjkYpZ6q6_tILru_GSnjuf2NmDMmg$) requires authors to make all data underlying the findings described in their manuscript fully available without restriction, with rare exception (please refer to the Data Availability Statement in the manuscript PDF file). The data should be provided as part of the manuscript or its supporting information, or deposited to a public repository. For example, in addition to summary statistics, the data points behind means, medians and variance measures should be available. If there are restrictions on publicly sharing data—e.g. participant privacy or use of data from a third party—those must be specified.

Reviewer #1: Yes

Reviewer #2: Yes

5. Is the manuscript presented in an intelligible fashion and written in standard English?

PLOS ONE does not copyedit accepted manuscripts, so the language in submitted articles must be clear, correct, and unambiguous. Any typographical or grammatical errors should be corrected at revision, so please note any specific errors here.

Reviewer #1: Yes

Reviewer #2: Yes

6. Review Comments to the Author

Please use the space provided to explain your answers to the questions above. You may also include additional comments for the author, including concerns about dual publication, research ethics, or publication ethics. (Please upload your review as an attachment if it exceeds 20,000 characters)

Reviewer #1: The authors’ explanation of the interpretation of CEMP makes more sense in this revision, but CEMP is rarely interpreted in the results section, which limits the readability and interpretability of the findings for readers. For example, on page 12 the authors point out that CEMP levels exceed 70% for ages 50-59 but do not interpret that this means that there are 70% more natural deaths than would be expected due to COVID for this age group (under the assumption that COVID deaths occurred only among those who otherwise would not have died).

**Reply:** We edited to add explanation of the CEMPs ratios.

The text states that “For Illinois, there is evidence of some undercounting of COVID-19 deaths: natural-non-COVID deaths rise during peak COVID-mortality periods (Figure App-4, Panel C)” and notes that this implies CEMP will be underestimated. There is evidence that mortality rates from several causes of death increased due to medical disruptions during the pandemic, so how can we be sure that these peaks imply undercounts of COVID-19 and not indirect effects of the pandemic?

**Reply:** We can’t be: rephrased. Both in this section (around page 8 in our draft) and later, in the limitations section, we added medical care disruptions during peak-COVID periods, which could affect non-COVID mortality, as an additional reason why CEMP could be underestimated.

How does a real change in non-COVID natural mortality that does not reflect undercounting impact the validity of the CEMP measure?

**Reply:** CEMP will be underestimated, either way. We can hope, but not prove, that the undercounting will be similar across racial/ethnic groups, so will not strongly affect CEMPS ratios. This is stated both in the section you commented on and in the limitations section.

I appreciate that the authors have substantially expanded their supplementary analyses to demonstrate the robustness of their findings in response to the reviewer comments. However, with the text now mentioning all these additional tables, it is harder for readers to follow. For example, the paragraph on page 7 starting with “We can validate...” describes three appendix tables in quick succession and readers may not grasp on first read how these support the CEMP measure. I would recommend revising/reorganizing the description of the appendix tables to not disrupt the flow of the paper so much.

**Reply:** We rewrote and expanded this discussion, to make it easier to follow.

Figure 1 with rates instead of absolute counts is much improved.

**Minor:**

Typo: The text (page 6) still describes excluding immune-compromised individuals.

**Reply**: Fixed; thank you for catching this.

Page 16: What is “2H 2021” for Omicron Period?

**Reply:** Edited, “Second Half of 2021”

Table 3 needs to have informative category labels for the age group rather than bins. I see the bins are defined in the caption, but this seems unnecessary when they could just be in the table.

**Reply:** We reorganized this table, so that the age ranges appear on separate rows for the younger and older groups.

Reviewer #2: All comments have been addressed. I have no further comments other than a few more typos that I have found.

Page 18, line 4. Should be Appendix Table (is currently Rable), and there are two periods at the end of this sentence.

**Reply:** Fixed.

While revising your submission, please upload your figure files to the Preflight Analysis and Conversion Engine (PACE) digital diagnostic tool, [https://pacev2.apexcovantage.com/](https://urldefense.com/v3/__https:/pacev2.apexcovantage.com/__;!!Dq0X2DkFhyF93HkjWTBQKhk!VMtgmOTuZXfOTJyfmPrRsHuzsafg6d3zyLSUXmbAnWg4pf4wmEPATag1H0BWXCQjkYpZ6q6_tILru_GSnjufPrEsZZQ$). PACE helps ensure that figures meet PLOS requirements. To use PACE, you must first register as a user. Registration is free. Then, login and navigate to the UPLOAD tab, where you will find detailed instructions on how to use the tool. If you encounter any issues or have any questions when using PACE, please email PLOS at [figures@plos.org](mailto:figures@plos.org). Please note that Supporting Information files do not need this step.
